# Remarkable Species Diversity of the Leafhopper Genus Xestocephalus (Hemiptera: Cicadellidae: Aphrodinae) in Thailand

**DOI:** 10.3390/insects12060514

**Published:** 2021-06-01

**Authors:** Zonglei Liang, Christopher H. Dietrich, Wu Dai

**Affiliations:** 1 Key Laboratory of Plant Protection Resources and Pest Management, Ministry of Education, Entomological Museum, Northwest A & F University, Yangling 712100, China; zonglei@nwsuaf.edu.cn; 2 Illinois Natural History Survey, Prairie Research Institute, University of Illinois, Champaign, IL 61820, USA; chdietri@illinois.edu

**Keywords:** Auchenorrhyncha, morphology, taxonomy, distribution, fauna

## Abstract

**Simple Summary:**

Thailand, a country on the Indochinese peninsula in Southeast Asia, is part of a biodiversity hot spot that harbors a large number of endemic species. The insect fauna remains poorly known, but a recent bioinventory has provided many new specimens for study. We review the common, widespread leafhopper genus *Xestocephalus* from Thailand and provide detailed morphological descriptions (the main evidence distinguishing species) and distributions of all species. Seventeen species were recognized, including twelve new species and four species recorded in Thailand for the first time.

**Abstract:**

*Xestocephalus* Van Duzee is among the most common and widespread genera of Cicadellidae in the temperate and tropical regions of the world. In the present study, 205 specimens of the genus *Xestocephalus* were collected in Thailand, whereas only a single species of the genus was recorded previously using Malaise trap field sampling, studied by comparative morphology. Seventeen species were recognized, including twelve new species: *X. binarius* sp. nov., *X. chrysanthemum* sp. nov., *X. cowboyocreus* sp. nov., *X. densprint* sp. nov., *X. dimiprocessus* sp. nov., *X. exproiecturus* sp. nov., *X. gracilus* sp. nov., *X. limpidissimus* sp. nov., *X. malleus* sp. nov., *X. nonattribus* sp. nov., *X. recipinams* sp. nov., and *X. tenusis* Liang sp. nov. Four species were recorded in Thailand for the first time: *Xestocephalus abyssinicus* Heller and Linnavuori, *Xestocephalus asper* Linnavuori, *Xestocephalus ishidae* Matsumura, and *Xestocephalus toroensis* Matsumura. Detailed morphological descriptions of all 17 species are given; photographs of external habitus and male genitalia of the species from Thailand are provided. A checklist of species of the genus is also given, and a key to all Thailand *Xestocephalus* species is also provided.

## 1. Introduction

Thailand, located on the Indochinese peninsula in Southeast Asia, is part of a biodiversity hot spot that harbors a large number of endemic species, estimated to comprise a significant proportion of the total species known worldwide [1]. The rich diversity of insect taxa may be explained in part by the country’s widespread tropical seasonal forests and floristic composition. However, until recently, few studies of Thai insects have been conducted. A recent biodiversity inventory project focused on insects, led by scientists at the University of Kentucky (USA) and Queen Sirikit Botanical Garden (Thailand), yielded large numbers of specimens from protected areas throughout the country [2]. A previous study of these samples revealed numerous new and apparently endemic genera and species of many different insect groups, including leafhoppers [3,4,5,6,7,8].

Leafhoppers (Cicadellidae) comprise the largest family of Hemiptera and are one of the largest families of plant-feeding insects, with nearly 24,000 described species in over 2600 genera [9]. Recent investigations on leafhoppers in Thailand suggest that most of the species encountered in recent collecting remain undocumented [4,7].

*Xestocephalus*, first described by Van Duzee in 1892, is one of the larger leafhopper genera and one of very few distributed worldwide, with >180 previously described species (Appendix A). Adults are small to moderately large (2.0 to 5.0 mm long), ovoid, and usually yellow or brown with black or brown spots or fascia. The head is usually bluntly rounded anteriorly and possesses short antennae with a prominent antennal ledge. The crown is narrower than the pronotum, and the ocelli are located on the boundary between the crown and face. The forewing venation is complete, but the hind wing has the submarginal vein incomplete apically. Species occur in all biogeographic regions except the central parts of the Palaearctic and are known from a variety of habitats, ranging from tropical rainforests to temperate grasslands. Species of this genus are by far the most abundant leafhoppers in Oligo-Miocene Dominican amber [10] but have not been documented in earlier fossil faunas. The extant species feed and breed on many kinds of vegetation, including herbs, grasses, and sedges. In some grassland species, the adults live close to the soil surface [11,12], and nymphs may live in the soil [13] or feed on grass roots [12,14]. Several species in the genus are known to transmit plant pathogens, and all are capable of inflicting injury to plants [15,16,17].

Phylogenetic relationships of *Xestocephalus* remain poorly investigated. The classification system is currently based on differences in the shape and ornamentation of the aedeagus and styles in the male genitalia. An intuitive morphology-based phylogeny of North and Central American species, including the West Indies, grouped species into seven lineages based on male genitalia characters, with individual species distinguishable based on discrete differences in the shape of the aedeagus and style and in coloration [18].

Reviews of the species of *Xestocephalus* from Canada and Alaska [11], North and Central America [18], Central and South America [19,20,21,22,23], Africa [24], Madagascar [25], Australia [26], New Zealand [26,27], the Indian subcontinent [28,29], Japan [30,31,32], and China [33,34,35] have resulted in a better understanding of the diversity and distribution of the species, but the fauna of Southeast Asia remains poorly studied.

New species of *Xestocephalus* continue to be discovered [23], but only a few previous papers have dealt with the Asian fauna, and only nine nominal species have previously been described or recorded in Southeast Asia [36]. Only one species, *X. guttatus,* was previously recorded in Thailand [37].

This study reports the findings of our studies of recently collected material from Thailand and provides new morphological and distributional data for the country based on fine-scale field sampling and analysis of 205 specimens from several provinces throughout the country. A world checklist of *Xestocephalus* species is also provided (Appendix A). Our results will facilitate further research aimed at understanding phylogenetic and distributional patterns for this large, cosmopolitan genus.

## 2. Materials and Methods

The specimens examined were collected using Malaise traps and are deposited in the collections of Queen Sirikit Botanic Gardens, Chiang Mai, Thailand (QSBG); the Illinois Natural History Survey, Champaign, IL, USA (INHS); and the Entomological Museum of Northwest A&F University (NWAFU), Yangling, Shaanxi, China.

The identification of specimens was based mainly on morphological comparisons of male genitalia. Abdomens were removed from specimens and soaked in 10% NaOH for 8 h to dissolve the muscle, washed in distilled water, and then transferred to glycerin for further dissection and examination. Digital micrographs were taken using an OLYMPUS PM-10AD (Olympus Co. LTD, Tokyo, Japan) and a Nikon AFX-II stereomicroscope (Nikon Imaging Japan Inc., Tokyo, Japan) with a Q Imaging digital camera (QImaging, Surrey, BC, Canada), captured in Q-Capture Pro 7 (QImaging, Surrey, BC, Canada) and compiled in Auto-Montage Pro (Synoptics Ltd., Cambridge, UK). Photographs were modified with Adobe Photoshop CS2 (Adobe Systems, San Jose, CA, USA).

Morphological terminology used in this study follows Cwikla [18], as modified by Rakitov [38] and Dietrich [39]. This published work and the nomenclatural acts it contains have been registered in ZooBank, the online registration system for the ICZN (International Code of Zoological Nomenclature). The LSID (Life Science Identifier) for this publication is: urn:lsid:zoobank.org:pub:844EF3D2-5845-4FFD-A949-A4E43102C61C.

## 3. Results and Discussion

### 3.1. Generic Characters

Family Cicadellidae Latreille 1825

Subfamily Aphrodinae Haupt 1927

Tribe Xestocephalini Baker 1915

*Xestocephalus* Van Duzee, 1892

*Xestocephalus* Van Duzee, 1892: 298 [40]. Type Species: *Xestocephalus pulicarius* Van Duzee, 1894 [41], by subsequent designation of Distant, 1908g [28].

**Redescription**. (Modified from Linnavuori (1959) [19], Cwikla (1985) [18], Kamitani (1996, 2005) [31,32].

Small and robust, body oblong, 2.5–5.0 mm in length. Coloration usually brown or dark brown with patterns variously developed and consisting of pale or dark lines or spots, symmetrically arranged. Face, leg including setae, and abdomen concolorous with rest of body.

Head includes eyes narrower than pronotum. Crown bluntly rounded in front; crown texture completely shagreen, vertex shorter than width between eyes, smoothly rounded to face in lateral aspect. Ocelli situated on boundary between vertex and frontoclypeus, closer to midline than to eye. Coronal suture indistinct. Eyes grayish, in line with lateral margins and emarginated next to antennae. Face convex, slightly wider than long, only slightly sinuate laterally beneath eyes. Lateral frontal sutures distinct, extended dorsomesad from antennal pits and terminating just ventromesad of ocelli. Antennal pits relatively deep. Clypellus short, relatively broad, constricted near base. Frontoclypeus expended laterally at the level of antennal pits. Pronotum carinate, wider than long, lateral margins moderately long, posterior margin shallowly concave medially. Forewing subhyaline, with 3 closed subapical and 4 apical cells; appendix very narrow or apparently absent; forewing apices not or only slightly overlapping at rest. Hind wing with submarginal vein evanescent apically. Fore femur ventrally with a single long macrosetae situated at midlength, usually with single short stout seta along basal 2/3 of row AV; fore tibia AD and PD each with 1 distal macroseta. Hind femur setal formula 2 + 1 + 1.

Male genitalia. Pygofer moderately produced posteriorly; posterior margin varying from narrowly to broadly rounded; macrosetae distributed over posterior half, lobe often with a short internal process. Valve transverse, much wider than long, with anterior and posterior margin parallel, or with posterior margin roundly produced, not tapered or pointed. Subgenital plate narrow at base, linguiform or triangular, bearing macrosetae and many fine setae. Style S-shaped, apophysis long, apex enlarged and boot-shaped, pre-apical lobe roundly produced laterad. Connective somewhat cruciate, arms short. Aedeagus with base U-shaped in lateral view, with or without appendages. Gonopore on posteroventral surface.

Female genitalia. Abdominal sternite VII in ventral view variable interspecifically; anterior margin straight or slightly produced anteriorly; posterior margin with median area slight concave, with shallow concavity or V-shaped median notch. Ovipositor distinctly arched with distal half curved downward. First valvulae in lateral view broadly expanded in apical half and tapered preapically to attenuate apex, dorsal sculpture consisting of single narrow submarginal row of short, curved, vertical strigae extended over most of length, ventral margin with short apical area of baculiform sculpture. Second valvulae in lateral aspect broad, parallel-sided through most of length, dorsal margin without teeth or serrations, ventral margin with preapical angulate prominence. Third valvulae very narrow through most of length, abruptly broadened preapically, with sparse small setae ventrally.

**Distribution.** The genus *Xestocephalus* is distributed worldwide, with the exception of Europe.

**Notes.***Xestocephalus* was erected by Van Duzee [40], and *Xestocephalus pulicarius* was designated as the type of the genus by Distant [28]. Lindberg [42] described *Nesotettix* from the Canary Islands, but it was a homonym of *Nesotettix* Holdhaus [43] and replaced with *Lindbergana* by Metcalf [44]. Linnavuori [19] treated *Lindbergana* as a junior synonym of *Xestocephalus* in his revision of the New World species.

Kirkaldy [45] regarded *Xestocephalus* as a relative of *Phrynomorphus* Curtis (*Euscelis* Brullé). Oman [46], Evans [25,47] and Linnavuori [48,49] placed Xestocephalini as a tribe of Deltocephalinae. Later, based on differences in the form of the anterior tentorium branches, the plesiomorphic structure of the male genitalia, and leg chaetotaxy, Oman [13] and Linnavuori [19,50] regarded Xestocephalinae as a distinct subfamily. Hamilton [51,52] placed *Xestocephalus* Van Duzee and related genera in subtribe Xestocephalina of Aphrodini, which was placed within his broad circumscription of Aphrodinae, but few subsequent authors have followed this classification. Linnavuori [24] suggested that *Xestocephalus* was derived from a common ancestor with other Aphrodinae s.s. during the Tertiary period. *Xestocephalus* was most recently included in Aphrodinae (Xestocephalini) based primarily on the characteristics of leg chaetotaxy and male genitalia [39].

*Deltocephalus guttulatus* Motschulsky, 1859 [53] is the first described species referable to *Xestocephalus*. Later, Berg [54] described the North American species *Athysanus desertorum*, which is considered a senior synonym of the type species *X. pulicarius*. This same species, which varies in coloration, was described as *Deltocephalus superbus* by Provancher [55]. *Xestocephalus* was originally defined mainly on external characteristics and may be easily differentiated from other Aphrodinae by the relatively small size, head with crown rounded to face, ovate frontoclypeus expanded laterally over the antennal base and narrower than the pronotum, and the dorsum usually with many round pale spots. Previously, the characteristics used to separate species were chiefly the markings on the vertex and pronotum or, in species without such markings, the general color of the vertex and the markings of the forewing [56]. Later, the aedeagus and styles of the male genitalia and the pygofer internal process were regarded as key characteristics for classifying *Xestocephalus* species, although they were shown to vary intraspecifically in some cases [57,58]. A comparative study of the male genitalia of available specimens revealed that variation occurs in the structure of the style apophysis and preapical lobe, the internal process of the pygofer, the shape of the aedeagal shaft, and the position of the gonopore. In some groups, the aedeagus shows little variation, but the style apophysis and internal process are slightly different, which resulted in the recognition of these variants as different species [31,58] or subspecies [24]. In other cases, subspecies have been recognized based on slight differences in the aedeagus [50] or coloration [49]. The structure of the aedeagus was used to estimate the phylogenetic relationships of the species of North and Central America, including the West Indies, by Cwikla [18], who divided this fauna into seven species groups. Unfortunately, no further studies of the phylogeny of *Xestocephalus* have been attempted.

The biology of the tribe Xestocephalini is poorly understood. At least some species are myrmecophiles, and the nymphal stage is apparently subterranean [24]. Rakitov [14] showed that neither nymphs nor adults of the widespread American species *X. desertorum* appear to be associated with ant nests. Nymphs of this species feed on the roots of grasses at or slightly below the soil surface, and adults feed on both grass roots and other parts of the grass. Eggs are laid singly on the surface of grass roots. After each molt, both nymphs and adults use their legs to cover the fresh integument with droplets exuded from the anus, containing brochosomes, secretory particles produced in the Malpighian tubules [14].

So far, there have been few revisionary studies of *Xestocephalus* species in the Oriental region. The scattered published records of species are from China [31,32,34,35,59,60], India [28,29,61], Indonesia, Malaysia [36,62], Philippines [61,63], and Thailand [37]. Further investigations with more taxonomic sampling are needed to reveal the biodiversity of *Xestocephalus* in the Oriental region.

### 3.2. Overview of Species Identification, Distribution

Seventeen species of the leafhopper genus *Xestocephalus* from Thailand are recorded based on a comparative morphological study, including twelve new species and four new recorded species, as shown in the following section (Figure 1, Figure 2, Figure 3, Figure 4, Figure 5, Figure 6, Figure 7, Figure 8, Figure 9, Figure 10, Figure 11, Figure 12, Figure 13, Figure 14, Figure 15, Figure 16, Figure 17, Figure 18, Figure 19, Figure 20, Figure 21, Figure 22, Figure 23, Figure 24, Figure 25, Figure 26 and Figure 27).

#### 3.2.1. Checklist of the Genus *Xestocephalus* from Thailand

*Xestocephalus abyssinicus* Heller and Linnavuori, 1968, n. rec. Thailand (Prachuab), Ethiopia.

*Xestocephalus asper* Linnavuori, 1969, n. rec. Thailand (Chaiyaphum, Chiang Mai, Nakhon Nayok), Congo, Sudan, Guinean.

*Xestocephalus gracilus* sp. nov. Thailand (Kamphaeng Phet).

*Xestocephalus binarius* sp. nov. Thailand (Chiang Mai).

*Xestocephalus chrysanthemum* sp. nov. Thailand (Nakhon Si, Kamphaeng, Chiang Mai).

*Xestocephalus cowboyocreus* sp. nov. Thailand (Chiang Mai).

*Xestocephalus densprint* sp. nov. Thailand (Chiang Mai).

*Xestocephalus dimiprocessus* sp. nov. Thailand (Chiang Mai).

*Xestocephalus exproiecturus* sp. nov. Thailand (Phetchabun, Nakhon Nayok, Lampang, Chanthaburi).

*Xestocephalus guttulatus* (Motschulsky, 1859) Thailand (Phetchabun, Chiang Mai), Sri Lanka, Japan, Tanzania, Ethiopia, Taiwan, Malaysia, Indonesia (Java), Philippines, Korea, Russia, Turkey.

*Xestocephalus ishidae* Matsumura, 1914, n. rec. Thailand (Loei, Chaiyaphum, Phetchabun, Nakhon Ratchasima), Japan.

*Xestocephalus limpidissimus* sp. nov. Thailand (Lampang).

*Xestocephalus malleus* sp. nov. Thailand (Petchaburi).

*Xestocephalus nonattribus* sp. nov. Thailand (Chiang Mai, Suphanburi, Loei, Chaiyaphum, Prachuab, Nakhon Nayok, Kanchanaburi).

*Xestocephalus recipinams* sp. nov. Thailand (Nakhon Si, Petchaburi).

*Xestocephalus tenusis* sp. nov. Thailand (Chiang Mai, Chaiyaphum).

*Xestocephalus toroensis* Matsumura, 1914, n. rec. Thailand (Lampang, Nakhon Si, Kamphaeng, Loei), China, Japan.

#### 3.2.2. Key to Species of *Xestocephalus* from Thailand

1. Aedeagus without process.............................................................................................................................................................................................2
- Aedeagus with pair of processes at apex or base..........................................................................................................................................................6
2. Style without conspicuous preapical teeth; aedeagus with gonopore subapical on caudal margin………..........................................................3
- Style with conspicuous preapical teeth; aedeagus with gonopore near middle on caudal margin........................................................................5
3. Aedeagal shaft with longitudinal flange on either side of anterior margin, dorsal and ventral margins almost parallel (Figure 4F)...............
  ............................................................................................................................................................................................................*ishidae* Matsumura
- Aedeagal shaft without longitudinal flanges on anterior margin...............................................................................................................................4
4. Aedeagal shaft with anterior margin slightly curved anteriorly in lateral view (Figure 2F,H), tapering to apex in ventral view (Figure 2E,G)
  ...............................................................................................................................................................................................................*asper* Linnavuori
- Aedeagal shaft with anterior margin straight in lateral view (Figure 3F), broadened apically in ventral view (Figure 3E).................................
  ...................................................................................................................................................................................................*guttulatus* (Motschulsky)
5. Pygofer without internal process (Figure 7B); aedeagal shaft compressed (Figure 7E–G) in posterior view, robust in lateral view (Figure 7H–J)
  ...........................................................................................................................................................................................................*nonattribus* sp. nov.
- Pygofer with internal processes present (Figure 6B); aedeagal shaft columnar, slender in lateral view (Figure 6F)...................*gracilus* sp. nov.
6. Subgenital plates linguiform (Figure 20C); aedeagus with processes at base (Figure 20E,F)………………………….…………………………..7
- Subgenital plates triangular; aedeagal with processes at apex.................................................................................................................................12
7. Aedeagus with two pairs of basal processes (Figure 17E,F)...............................................................................*abyssinicus* Heller and Linnavuori
- Aedeagus with one pair of basal processes (Figure 20E, Figure 21E and Figure 22E).............................................................................................8
8. Aedeagal processes located on ventral base of shaft (Figure 22F and Figure 23F).................................................................................................9
- Aedeagal processes on apodeme (Figure 20E,F and Figure 21E,F)...........................................................................................................................10
9. Pygofer with triangular posteroventral processes, internal processes triangular (Figure 23B); aedeagal shaft straight, with processes slightly
  broadened at apex (Figure 23F).........................................................................................................................................................*malleus* sp. nov.
- Pygofer with longer dorsal processes and ventro-posterior processes, internal processes hook-like (Figure 22B); aedeagal shaft slightly curved
  anteriorly, with processes tapering to apex (Figure 22E,F)…………………….....................................................................*dimiprocessus* sp. nov.
10. Pygofer without processes on caudal margin (Figure 21B); style with prominent preapical heel, without teeth (Figure 21D); aedeagal
  apodeme swollen in lateral view (Figure 21F)........................................................................................................................*limpidissimus* sp. nov.
- Pygofer with processes on caudal margin (Figure 20B); style with prominent preapical heel, with teeth (Figure 20D); aedeagal apodeme
  smooth in lateral view (Figure 20F)..............................................................................................................................................................................11
11. Pygofer with two large triangular processes at posteroventral margin (Figure 20B); style with several subapical tooth-like processes on
  lateral margin (Figure 20D)..............................................................................................................................................................*recipinams* sp. nov.
- Pygofer with a long hook-like process at dorsoposterior margin (Figure 18B); style without tooth-like processes on lateral margin (Figure 18D)
  ..........................................................................................................................................................................................................*cowboyocreus* sp. nov.
12. Aedeagus with a pair of apical processes (Figure 9E,F)..................................................................................................................*binarius* sp. nov.
- Aedeagus with two pairs of apical processes (Figure 10E,F).....................................................................................................................................13
13. Aedeagal processes short, only 1/3 length of shaft (Figure 10E,F)..............................................................................................*densprint* sp. nov.
- One pair of aedeagal processes equal to 1/2 length of shaft......................................................................................................................................14
14. Aedeagus with upper processes shorter than lower processes (Figure 11E and Figure 12E)............................................................................15
- Aedeagus with upper processes almost equal to lower processes (Figure 14E and Figure 15E)..........................................................................16
15. Aedeagal processes slight curved anteriorly (Figure 12F), apical processes curved upper in caudal view (Figure 12E)...................................
  ................................................................................................................................................................................................*chrysanthemum* sp. nov.
- Aedeagal shaft straight (Figure 11F), apical processes almost straight in caudal view (Figure 11E)....................................*toroensis* Matsumura
16. Aedeagal shaft abruptly narrowed to distal third of shaft (Figure 15F), bases of two pairs of apical processes separated (Figure 15E,F).....
  .......................................................................................................................................................................................................*exproiecturus* sp. nov.
- Aedeagal shaft gradually tapering to apex (Figure 14F), bases of two pairs of apical processes linked (Figure 14E,F)...............*tenusis* sp. nov.

### 3.3. Species Descriptions

#### 3.3.1. *Xestocephalus asper* Linnavuori, 1969, n. rec.

**Figure 1:**
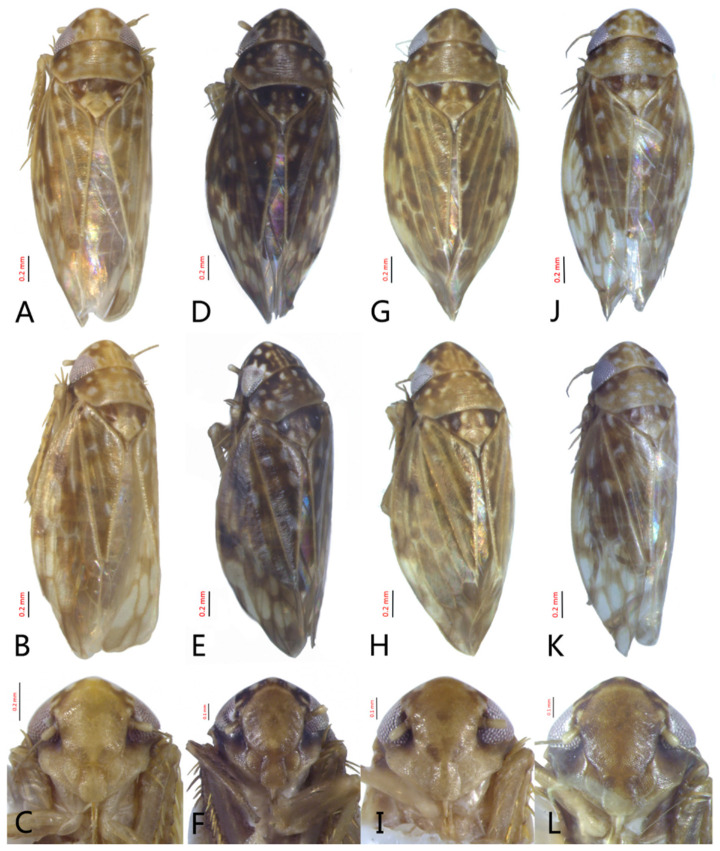
Male habitus of *Xestocephalus*. (**A**–**F**) *X. asper*; (**G**–**I**) *X. guttulatus*; and (**J**–**L**) *X. ishidae*. (**A**,**D**,**G**,**J**) dorsal view; (**B**,**E**,**H**,**K**) laterodorsal view; (**C**,**F**,**I**,**L**) face.

**Figure 2:**
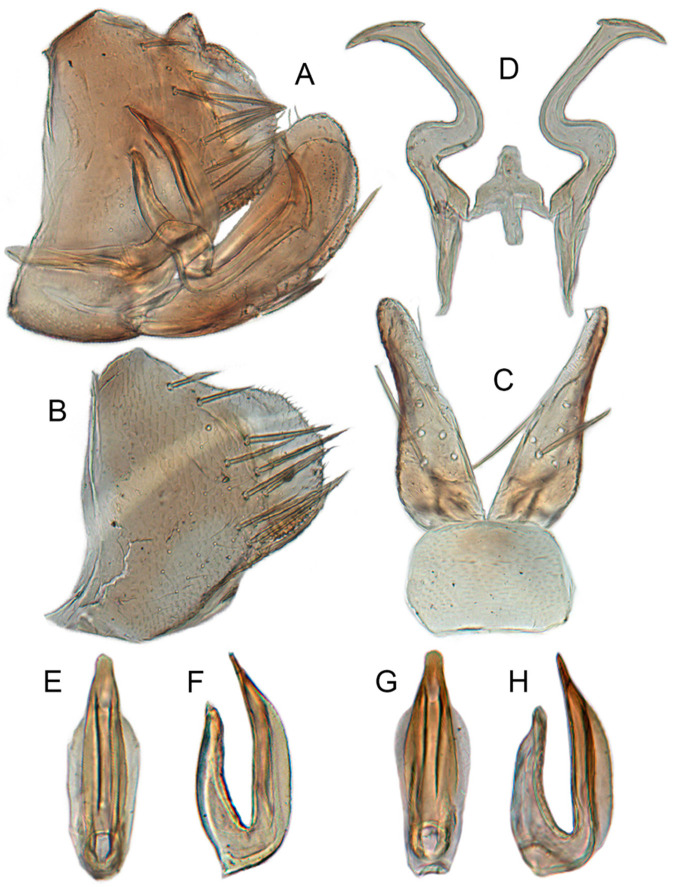
*Xestocephalus asper* Linnavuori, 1969. (**A**) Male genital capsule, lateral view; (**B**) pygofer lobe, lateral view; (**C**) subgenital plates and valve, ventral view; (**D**) style and connective, ventral view; (**E**,**G**) aedeagus, posterior view; (**F**,**H**) aedeagus, lateral view. (**A**–**F**) from Chaiyaphum and (**G**,**H**) from Chiang Mai.

Figure 1A–F, Figure 2A–F, Figure 24A, and Figure 25A1–A3.

*Xestocephalus asper* Linnavuori, 1969: 1149, Figure 16a–c [64]; Linnavuori, 1979: 934, Figure 49a–c [24].

**Redescription.** Length: ♂ 2.7–2.9 mm, ♀ 2.7–2.9 mm. Coloration includes two morphs: Morph 1 is dark brown with grayish-white markings (Figure 1D–F): Morph 2 is pale ochraceous with gray markings (Figure 1A–C). Vertex with three round grayish-white spots on anterior margin and four grayish-white spots on submargin, discal area with a grayish-white longitudinal line on each side of brown midline and narrower transverse grayish-white band posteriorly, a small grayish-white spot adjacent to basal angles of eyes. Face pale brown, with marginal arcuate band dark brown. Pronotum dark-brown, with several distinct cream spots at anterior margin and submargin. Scutellum dark-brown with two grayish-white irregular marginal spots on each side of anterior margin and one grayish-white marginal spot on each side of posterad of scutellar suture. Forewings with unpigmented spots pale. Ventral surface and legs uniformly dark brown.

**Male genitalia.** Pygofer in lateral view narrowed toward round caudal margin, slightly higher than long, posteroventral margin slightly curved inward, with a tooth-like process on inner surface arising on the ventral margin and extending posterodorsally, macrosetae arranged in preapical vertical band, posterior margin with many tiny setae. Valve transverse, much wider than long, with parallel anterior and posterior margin. Subgenital plate broad linguiform, apical margin rounded, apical 2/3 of lateral margin curved inward, with two rows of macrosetae. Style slender, S-shaped, apex enlarged with short, acute preapical heel, apical margin evenly convex, without teeth. Connective cross-shaped, lateral arms extended anterolaterad, median anterior lobe slender and longer than lateral arms. Aedeagus symmetrical, shaft slightly longer than atrium, anterior margin relatively straight in lateral view, anterior margin shallowly concave with pair of dentate flanges on each side, abruptly tapering apically in lateral view. Gonopore subapical on caudal margin.

**Female**. Sternite VII nearly twice as wide as long, posterior margin slightly concave with slight V-shaped medial notch. Second valvulae with preapical ventral angle obtuse. Third valvula with preapical emargination slightly concave.

**Material examined.** 2♂♂, THAILAND: Chaiyaphum, Pa Hin Ngam NP, Deciduous forest near stream, 15°40.232′ N 101°27.255′ E, 398 m, Malaise trap, 13–19.vi.2007, Katae Sa-nog & Buakaw Adnafai; 2♂♂, Chiang Mai, Doi Inthanon NP campground pond, 18°32.657′ N, 98°31.482′ E, 1200 m, Malaise trap, 1–8.vii.2006, Y. Areeluck; 1♂4♀♀, Nakhon Nayok, Khao Yai NP Nhong ping khaokeaw, 14°23.094′ N, 101°23.055′ E, 733 m, Malaise trap, 12–19.iii.2007, Wirat Sukho; 2♂♂, Chiang Mai, Doi Inthanon NP campground pond, 18°32.657′ N, 98°31.482′ E, 1200 m, Malaise trap, 1–8.vii.2006, Y. Areeluck (QSBG, INHS, NWAFU).

**Distribution.** Thailand (Chaiyaphum, Chiang Mai, Nakhon Nayok) (Figure 27), Congo, Sudan, Guinean.

**Remarks.***X. asper* was described by Linnavuori [64] based on one male and two female specimens from the Congo. Later, he also described *X. asper pseudoguttulatus* for a variant with a smaller body and blunter crown [24]. This species is similar to *X. subfusculus* Melichar but differs from the latter in having the lateral lamellae of the aedeagus narrow. Different specimens examined from Chiang Mai, Thailand, appear to correspond to both subspecies recognized by Linnavuori, but specimens from Chaiyaphum and Nakhon Nayok correspond only to the nominotypical subspecies. The aedeagus of specimens from Thailand differs slightly from specimens from Africa illustrated by Linnavuori [64] in having the lateral flange somewhat narrower in posterior view. We interpret this variation as intraspecific but a study of more specimens from both Africa and Southeast Asia will be necessary to determine the taxonomic significance of such variation.

#### 3.3.2. *Xestocephalus guttulatus* (Motschulsky, 1859)

**Figure 3:**
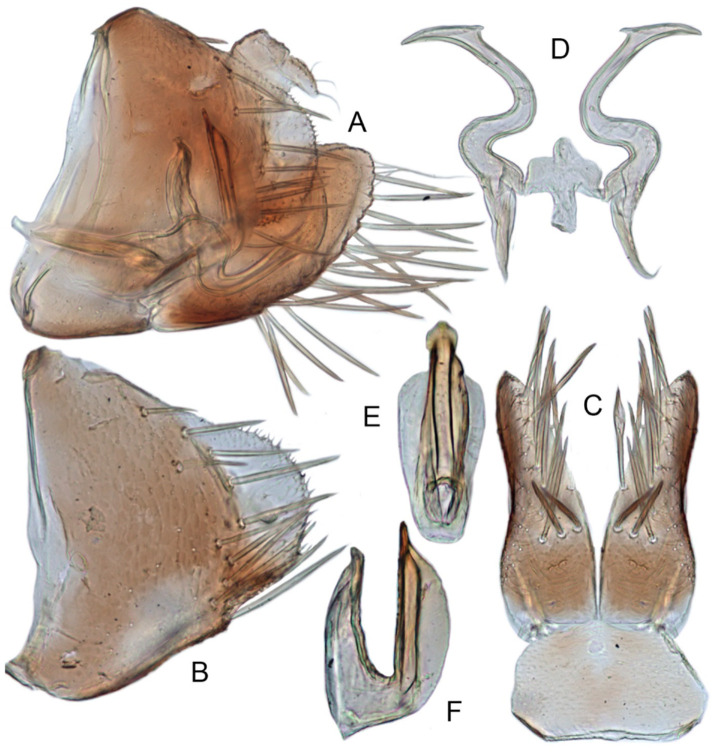
*Xestocephalus guttulatus* (Motschulsky, 1859), (**A**) Male genital capsule, lateral view; (**B**) pygofer lobe, lateral view; (**C**) Subgenital plates and valve, ventral view; (**D**) Style and connective, ventral view; (**E**) Aedeagus, posterior view; (**F**) aedeagus, lateral view.

Figure 1G–I, Figure 3A–F, Figure 24B and Figure 25B1–B3.

*Deltocephalus guttulatus* Motschulsky, 1859b: 113 [53].

*Deltocephalus guttatus* Motschulsky, 1863: 100 [65].

*Xestocephalus guttatus* Matsumura, 1902a: 403, Figure 29 [66]; Melichar, 1903b: 206, 207 [67]; Melichar, 1905: 303 [68]; Oshanin 1906a: 139 [69]; Distant, 1908g: 349, 350, Figure 221 [28]; Oshanin 1910: 166 [70]; Oshanin 1912a: 108 [71]; Matsumura 1914: 203, 204 [59]; Melichar 1914b: 138, 139 [62]; Nawa, 1914a: 190 [72]; Matsumura 1915a: 156, 182 [73]; Schumacher 1915a: 106 [74]; Schumacher 1915b: 126 [75]; China 1935b: 307 [76]; Zachvatkin 1935: 109 [77]; Merino 1936: 387, 396 [63]; Ishihara, 1953b: 25, Figure 2 [78]; Esaki and Ito 1954: 3, 85 [79]; Capco, 1960: 43 [61]; Ishihara, 1961a: 241 [37].

*Xestocephalus guttulatus* Metcalf, 1967c: 2363 [80]; Nast, 1972a: 241 [81]; Linnavuori, 1979b: 936, Figure 51f–h [24].

**Redescription.** Length: ♂ 2.3–2.5 mm, ♀ 2.8–2.9 mm. Light brown with numerous cream-colored patches and brown cloudy markings all over. Vertex brown with three small whitish spots, two ocelli respectively situated in the white dot on each side; two obscure short pale stripes next to each eye. Face brown and immaculate. Pronotum mottled with many irregular small pale spots; anterior margin, lateral angles ground-color brown. Scutellum mottled with two large diverging darker patches near basal angles, apical area lighter, with a crescent-like marking and neighboring four distinct cream-colored speckles. Forewings shiny light brown, with many elongate hyaline patches near apical portion and brown cloudy markings along costal margin; veins surrounded with shadows or whitish markings. Ventral surface and legs with uniform brown.

**Male genitalia.** Pygofer in lateral view higher than its length, furnished with approximately 12–15 macrosetae on posterior half, with caudal margin dentate and several distinct notches along the posteroventral margin. Internal process of pygofer small and obtuse-angled, directed ventrad. Valve short and trapezoidal. Subgenital plate moderately long, curved gradually and slightly dorsad, broadly rounded apically in lateral view, broad basally and then slightly narrowed to apex in ventral view, with two or three rows of macrosetae. Style slender, S-shaped, apical dilation of apophysis narrowly boot-shaped, without teeth. Connective cross-shaped, side arms folded anterad. Aedeagus with shaft straight, lightly longer than dorsal apodeme, slightly asymmetrical with longitudinal lamella on either side and apex expanded and diamond-shaped in ventral aspect. Gonopore subapical and ventral.

**Female genitalia.** Sternite VII posterior margin broadly V-shaped with narrow median notch. Second valvulae ventral preapical angle obtuse. Third valvula dorsal margin distinctly angulates preapically.

**Material examined.** 1♂3♀♀, THAILAND: Phetchabun, Nam Nao NP Pine forest/Sambon 1, 16°42.47′ N, 101°35.26′ E, 872 m, Malaise trap, 16–23.x.2006, Noopean Hongyothi; 2♂♂, Phetchabun, Nam Nao NP Hill evergreen forest, 16°44.402′ N, 101°34.56′ E, 883 m, Malaise trap, 26.v–2.vi.2007, Leng Janteab; 4♂♂, Chiang Mai, Doi Inthanon NP Vachirathan Fall, 18°32.31′ N 98°36.048′ E, 690 m, Malaise trap, 10–17.xi.2006, Y. Areeluck; 1♂, Chiang Mai, Doi Phahompok NP Headquarter, 19°57.961′ N, 99°9.355′ E, 569 m, Malaise trap, 25.vii–1.viii.2007, Wongchai.P. (QSBG, INHS, NWAFU).

**Distribution.** Thailand (Phetchabun, Chiang Mai) (Figure 27), Sri Lanka, Japan, Tanzania, Ethiopia, Taiwan, Malaysia, Indonesia (Java), Philippines, Korea, Russia, Turkey.

**Remarks.***X. guttulatus* was described by Motschulsky [53] as *Deltocephalus guttulatus* based on one male specimen from Sri Lanka. Matsumura [66] described the same species as *Xestocephalus guttulatus* based on one male and one female from Japan. Melichar [67] suggested that the latter is a synonym of the former but used the name *Xestocephalus guttatus*. After that, many authors included *Xestocephalus guttatus* in catalogues of Cicadellidae [28,37,59,61,62,63,66,67,68,69,70,71,72,73,74,75,76,77,78,79,81]. Linnavuori [24] redescribed this species and illustrated the male genitalia. The figures of male genitalia provided by Ishihara [30] and Anufriev and Emeljanov [82] indicate that these authors misidentified the species. *X. guttulatus* is morphologically very similar to *X. asper*, with only very slight differences in the aedeagus (e.g., shaft and dorsal apodeme broader in lateral view and lateral flange smooth rather than minutely serrate) and style (distal “foot” more elongated). Here, *X. guttulatus* and *X. asper* are tentatively retained as separate valid species, but a comparative study of additional specimens from throughout the range is needed to confirm their status.

#### 3.3.3. *Xestocephalus ishidae* Matsumura, 1914, n. rec.

**Figure 4:**
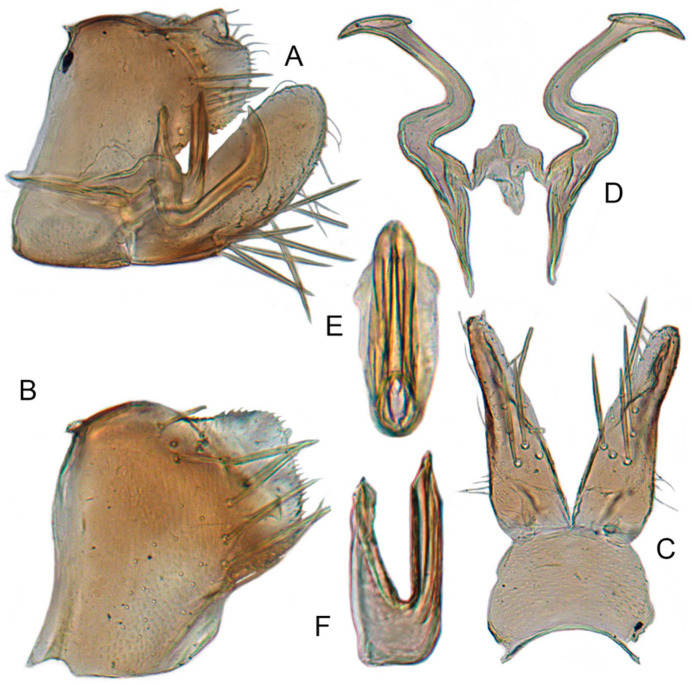
*Xestocephalus ishidae* Matsumura, 1914, n. rec. (**A**) Male genital capsule, lateral view; (**B**) pygofer lobe, lateral view; (**C**) subgenital plates and valve, ventral view; (**D**) style and connective, ventral view; (**E**) aedeagus, posterior view; (**F**) aedeagus, lateral view.

Figure 1J–L, Figure 4A–F, Figure 24C, and Figure 25C1–C3.

*Xestocephalus ishidae* Matsumura, 1914: 204 [59]; Ishihara, 1953b: 25 [78]; Ishihara, 1961b: 21 [30]; Kamitani, 2005: 27, Figures 25–30 [32].

**Redescription.** Length: ♂ 2.3–2.5 mm, ♀ 2.4–2.6 mm. Yellowish with numerous whitish cloudy markings. Crown relatively blunt, covered with several faint pale longitudinal stripes between eyes. Vertex dull yellow with a brownish irregular line extending from near apex to eye, curving around ocelli; two obscure whitish spots next to each eye. Face yellowish and immaculate. Pronotum mottled with two rows small pale spots; anterior margin, lateral angles ground-color fuscous. Scutellum mottled with two large diverging darker patches near basal angles, apical area lighter, with a crescent-like marking and neighboring four distinct pale speckles. Forewings shiny yellow throughout, with many elongate hyaline patches, especially along costal margin and apical portion; veins surrounded with shadows or whitish markings. Ventral surface and legs with uniform yellowish.

**Male genitalia.** Pygofer in lateral view higher than its length, narrowed toward round caudal margin with many macrosetae on posterior margin, caudal margin dentate with several distinct notches; process tooth-like and directed ventrad. Valve short and trapezoidal. Subgenital plate broad, linguiform, apical portion rounded, lateral margins of apex 2/3 slightly curved inwards, with two rows of macrosetae. Style slender, S-shaped, apex enlarged with prominent preapical heel, curved laterad, without teeth, preapical lobe developed. Aedeagus with shaft short, slightly longer than preatrium, slightly concave with an indistinct elongate flange on each side of anterior surface straight in lateral view, posterior margin nearly straight in lateral view with a slight flange on each side, abruptly narrowed apically, apical margin round in caudal view. Gonopore subapical on caudal margin.

**Female genitalia.** First valvulae wrinkle and ventral margin with serration. Second valvulae with densely fine striate along arcuate dorsal margin arcuate, with fine reticulate texture at apex.

**Material examined.** 1♂, THAILAND: Loei, Phu Kradueng NP Mixed deciduous/S Na Noy office, 16°49.01′ N, 101°47.62′ E, 276 m, Malaise trap, 21–28. v. 2008, Thonghuay Phatai; 2♂♂, Chaiyaphum, Pha Hin Ngam NP Dry Evergreen/Tepa waterfall, 15°33.88′ N 101°25.84′ E, 605 m, Malaise trap, 13–19.iv.2007, Katae Sa-nog and Buakaw Adnafai; 1♂5♀♀, Phetchabun, Khao Kho NP deciduous forest at Ta Pol river, 16°32.539′ N, 101°2.483′ E, 242 m, Malaise trap, 12–19. v. 2007, Somchai Chachumnan and Saink Singtong; 1♂, Nakhon Ratchasima, Khao Yai NP Moist evergreen forest at Dong Suer Paan, 14°27.511′ N, 101°22.408′ E, 760 m, Malaise trap, 19–23.xii.2006, Pong Sandow.

**Distribution.** Thailand (Loei, Chaiyaphum, Phetchabun, Nakhon Ratchasima) (Figure 27), Japan.

**Remarks.***X. ishidae* was described by Matsumura [59] based on one male specimen from Japan. Kamitani [32] examined the holotype and redescribed it, illustrated the external habitus, and was the first to illustrate the male genitalia. The examined specimens from Thailand are slightly different from the holotype in having the aedeagal shaft slightly concave on the anterior margin with two shallow flanges. This species is similar to *X. kuyanianus* Matsumura [32] but distinguished by aedeagal shaft straight, head and thoracic nota with markings, and with 8-10 macrosetae on the genital plate (aedeagal shaft slight curved, head and thoracic nota without markings, and with 20 macrosetae on the genital plate in *X. kuyanianus*).

#### 3.3.4. *Xestocephalus gracilus* sp. nov.

**Figure 5:**
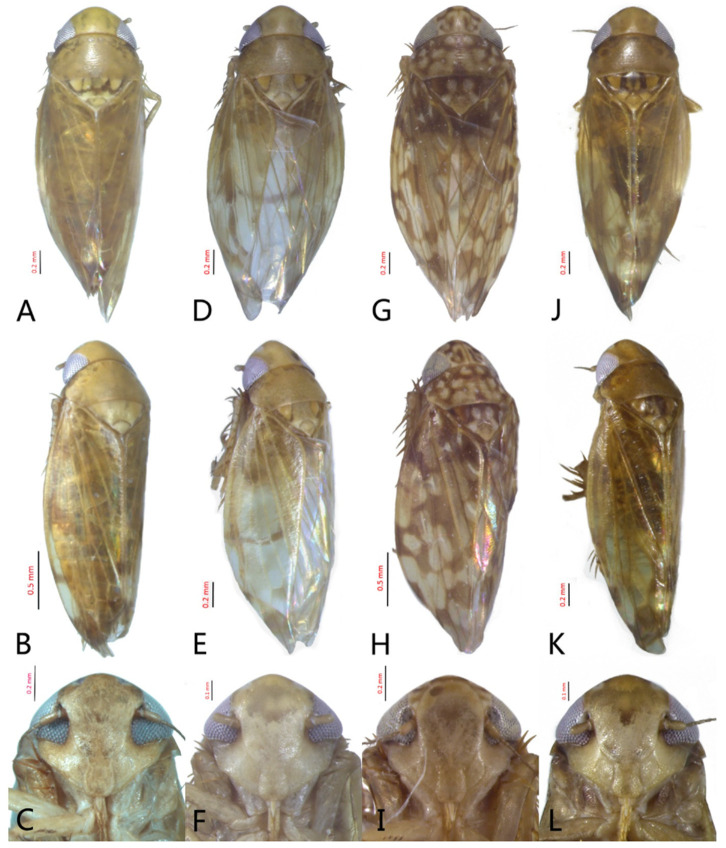
Male habitus of *Xestocephalus*. (**A**–**C**) *X. gracilus* sp. nov.; (**D**–**L**) *X.* *nonattribus* sp. nov. (**A**,**D**,**G**,**J**) dorsal view; (**B**,**E**,**H**,**K**) laterodorsal view; (**C**,**F**,**I**,**L**) face.

**Figure 6:**
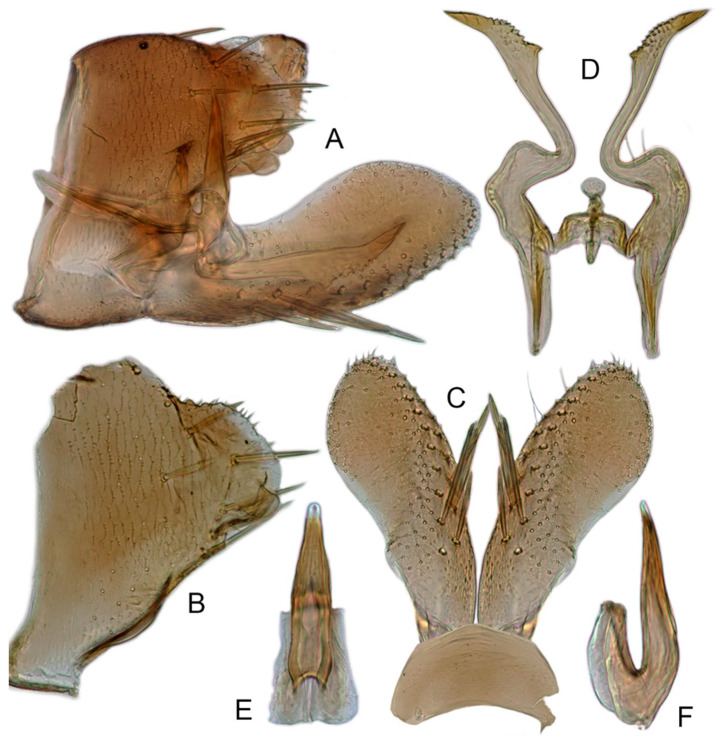
*Xestocephalus gracilus* sp. nov. (**A**) Male genital capsule, lateral view; (**B**) pygofer lobe, lateral view; (**C**) subgenital plates and valve, ventral view; (**D**) style and connective, ventral view; (**E**) aedeagus, posterior view; (**F**) aedeagus, lateral view.

Figure 5A–C and Figure 6A–F.

**Description.** Length: ♂ 2.9 mm. Crown, vertex, face, and pronotum yellowish, ocelli whitish. Scutellum mottled with a crescent-like marking on anterior angle. Forewings shiny yellow and hyaline. Ventral surface and legs with uniform faint yellow.

**Male genitalia.** Pygofer in lateral view higher than its length, narrowed to round caudal margin, with approximately 8 macrosetae on posterior half; internal process bluntly rectangular and directed ventrad. Valve short and trapezoidal. Subgenital plate broad, linguiform, apex rounded, lateral margins of apical 2/3 slightly curved inwards, with 2 rows of macrosetae. Style slender, S-shaped, with apical dilation of apophysis large thorn-shaped, with numerous scattered marginal and submarginal teeth, tapered to a point, apical 1/2 bent. Aedeagus with dorsal apodeme 1/2 times as long as shaft, shaft moderately long, with anterior margin straight in lateral view, tapering to apex gradually. Gonopore ventral, situated near basal 1/2 of shaft.

**Female.** Unknown.

**Material examined.** Holotype ♂, THAILAND: Kamphaeng Phet, Mae Wong NP Chong Yen, 16°5.212′ N 99°6.576′ E, 1306 m, Malaise trap, 17–24.iii.2008, Piluek C (QSBG).

**Distribution.** Thailand (Kamphaeng Phet) (Figure 27).

**Etymology.** The specific epithet is a Latin adjective referring to the slender aedeagus.

**Remarks.** The new species is similar to *X. punctulatus* Carvalho and Cavichioli [83] but can be distinguished from the latter by pygofer with internal process bluntly rectangular, the straight aedeagus with robust base, and the denticuli on the subapex of the styles (pygofer with internal process triangular, the straight aedeagus slight robust at middle in *X. punctulatus*).

#### 3.3.5. *Xestocephalus nonattribus* sp. nov.

**Figure 7:**
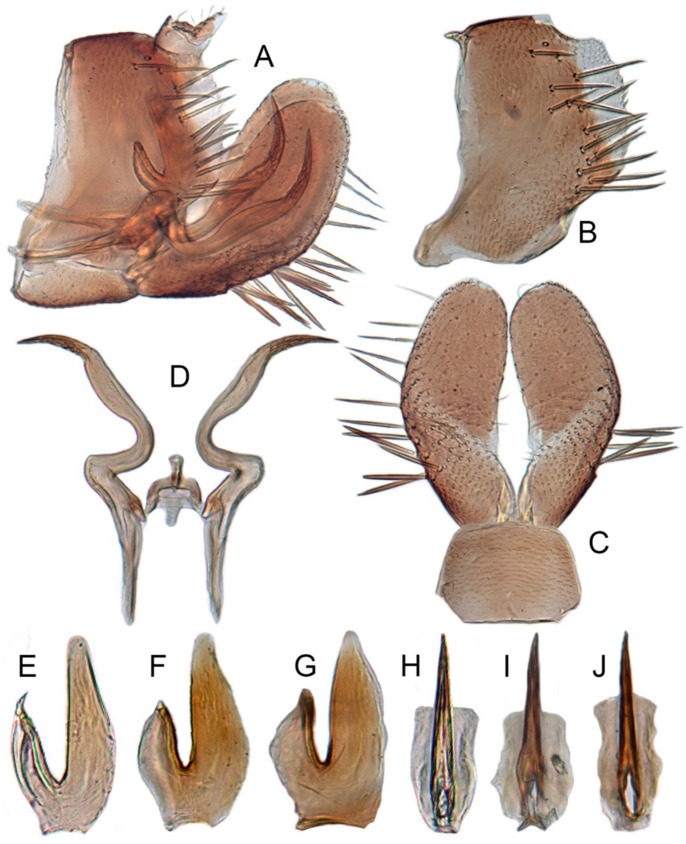
*Xestocephalus nonattribus* sp. nov. (**A**) Male genital capsule, lateral view; (**B**) pygofer lobe, lateral view; (**C**) subgenital plates and valve, ventral view; (**D**) style and connective, ventral view; (**E**–**G**) aedeagus, lateral view; (**H**–**J**) aedeagus, posterior view.

Figure 5D–L, Figure 7A–J, Figure 24D–F, and Figure 25D1–F3.

**Description.** Length: ♂ 2.6–2.8 mm, ♀ 2.7–2.8 mm. Color pattern with two different morphs, female color darker than male; one generally yellowish except forewings with brown cloudy markings at apex (Figure 5D–F,J–L); the other light brown with numerous pale patches all over dorsum (Figure 5G–I). Latter morph has crown covered with several pale obscure stripes between eyes. Vertex with a brownish irregular line extending from near apex to eye, curving around ocelli. Face yellowish and immaculate. Pronotum brown, with many irregular small pale spots evenly distributed. Scutellum brown, apical area light, with crescent-like marking and two distinct dumbbell-shaped pale spots. Forewings shiny brown, evenly covered with many elongate hyaline patches; veins surrounded with shadows. Ventral surface and legs uniform light yellow.

**Male genitalia.** Pygofer in lateral view higher than its length, with many macrosetae on posterior margin, internal process absent. Valve short and trapezoidal. Subgenital plate extending posteriorly farther than pygofer apex, broad, linguiform, basal half horizontal and apical half vertical, with apices broadly rounded in lateral view, with a row of macrosetae and serially arranged lateral microsetae. Style slender, S-shaped, with subapical part of apophysis slightly broadened, without preapical heel; elongated and tapered gradually to apex, texture of apical part irregularly reticulate. Connective cross-shaped, the side arms folded downward. Aedeagus with preatrium developed, shaft strongly compressed, slightly longer than preatrium, anterior margin straight, posterior margin rounded in profile, narrowed to rounded apex in lateral aspect, acuminate in posterior view. Gonopore slit-like near basal 1/3 of shaft.

**Female.** Sternite VII posterior margin somewhat variable truncate or broadly concave with acute median notch. Second valvulae with preapical angle 90°. Third valvula dorsal margin deeply concave preapically.

**Material examined.** Holotype ♂, THAILAND: Chiang Mai, Doi Phahompok NP Headquarter, 19°57.961′ N, 99°9.355′ E, 569 m, Malaise trap, 1–7.viii.2007, Wongchai. P. (QSBG). Paratypes: 12♂♂10♀♀, Suphanburi, Pu Toei NP *Pinus merkusii* forest, 14°58.4′ N, 99°26.017′ E, 763 m, Malaise trap, 1–7.ix.2008, Wangkum;P.; 2♂♂1♀, Suphanburi, Pu Toei NP Huai Mongpae/stream, 14°56.981′ N, 99°26.733′ E, 300 m, Malaise trap, 1–7.vii.2008, Saunbua.L.; 2♂♂1♀, Suphanburi, Pu Toei NP Huai Mongpae/red road, 14°56.985′ N, 99°26.78′ E, 300 m, Malaise trap, 24–31.vii.2008, Saunbua.L.; 2♂♂, Loei, Phu Kradueng NP Mixed deciduous/Elerd, 16°56.57′ N, 101°49.04′ E, 273 m, Malaise trap, 12–19.iii.2008, Thonghuay Phatai; 2♂♂1♀, Loei, Phu Ruea NP office, 17°28.826′ N, 101°21.33′ E, 860 m, Malaise trap, 12–19.vii.2006, Patikhom Tamtip; 1♂1♀, Loei, Phu Ruea NP office, 17°28.826′ N, 101°21.33′ E, 860 m, Malaise trap, 26.vii–2.viii.2006, Nukoonchai Jaroenchai; 1♂1♀, Chaiyaphum, Pa Hin Ngam NP creek at Tung Dok Grajeaw, 15°38.391′ N, 101°23.609′ E, 750 m, Malaise trap, 24–30.vii.2006, Kratae Sa-nog and Buakaw Adnafai; 1♂, Prachuab, Khiri Khan Khao Sam Roi Yot NP Nursery, 12°7.58′ N, 99°57.478′ E, Malaise trap, 10–17.viii.2008, Yai and Amnad; 2♂, Nakhon Nayok, Khao Yai NP near Training Center 2, 14°24.515′ N, 101°22.432′ E, 750 m, Malaise trap, 19–26.ii.2007, Wirat Sukho; 1♂1♀, Kanchanaburi, Khuean Srinagarindra NP Tha Thung-na/Chong Kraborg, 14°29.972′ N, 98°53.035′ E, 210 m, Malaise trap, 5–12.iii.2009, Boonnam and Phumarin (INHS, NWAFU).

**Distribution.** Thailand (Chiang Mai, Suphanburi, Loei, Chaiyaphum, Prachuab, Nakhon Nayok, Kanchanaburi) (Figure 27).

**Etymology.** The species name is a Latin adjective, which refers to the aedeagal shaft without processes.

**Remarks.** Comparison of male specimens from different localities in Thailand suggests that this species is somewhat variable in coloration and male genitalia. The discrepancy in color may be a result of preservation in alcohol, but specimens from different localities also exhibit slight variations in the shape of the aedeagus in lateral view (cf. Figure 7E,F). These forms intergrade, so we consider them to belong to a single species.

The new species is similar to *X. medius* Linnavuori in aedeagus but distinguished by the apically slender apophysis of the styles (thickened apophysis in *X. medius*).

#### 3.3.6. *Xestocephalus binarius* sp. nov.

**Figure 8:**
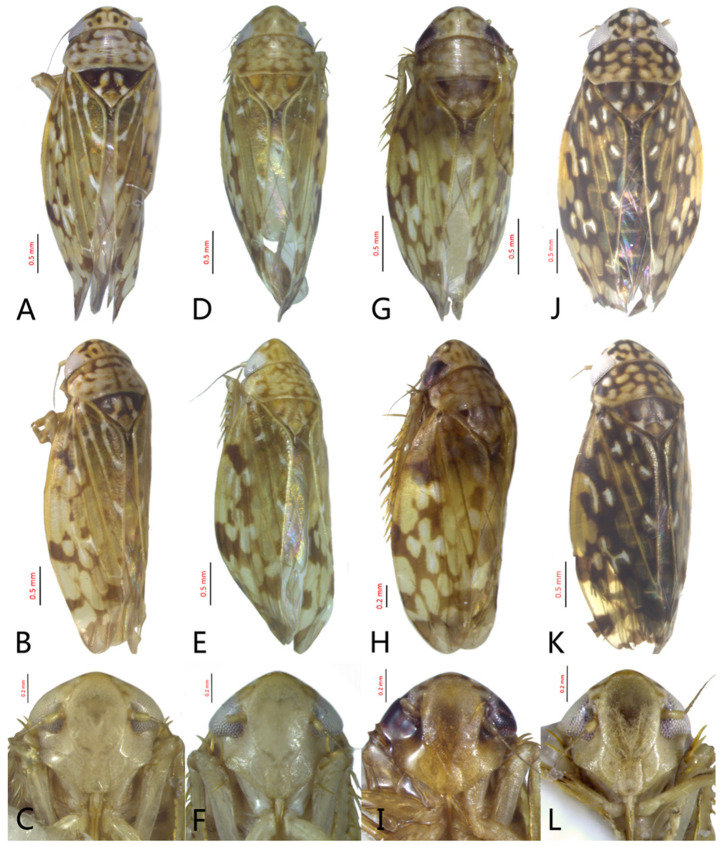
Male habitus of *Xestocephalus*. (**A**–**C**) *X.* *binarius* sp. nov.; (**D**–**F**) *X.* *densprint* sp. nov.; (**G**–**I**) *X.* *toroensis*; (**J**–**L**) *X.* *chrysanthemum* sp. nov. (**A**,**D**,**G**,**J**) dorsal view; (**B**,**E**,**H**,**K**) laterodorsal view; (**C**,**F**,**I**,**L**) face.

**Figure 9:**
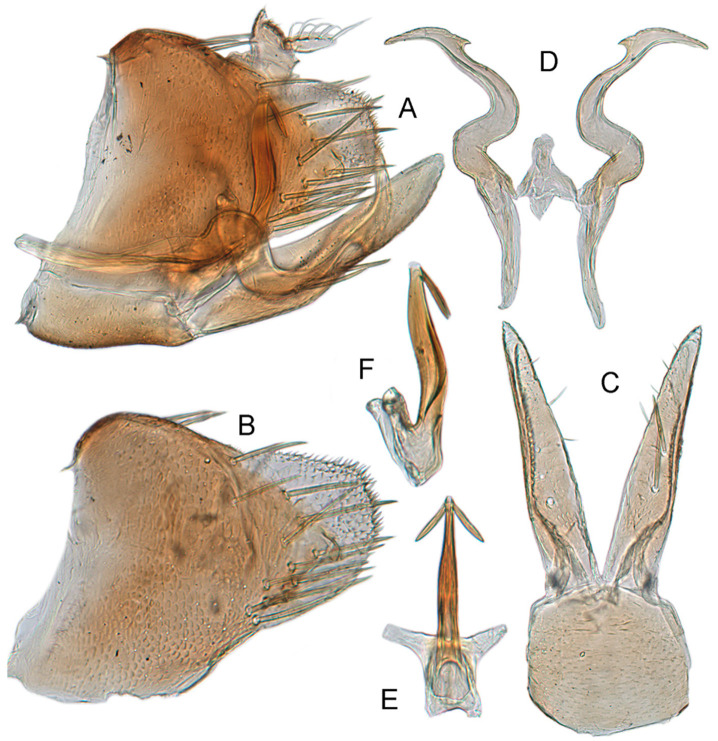
*Xestocephalus binarius* sp. nov. (**A**) Male genital capsule, lateral view; (**B**) pygofer lobe, lateral view; (**C**) subgenital plates and valve, ventral view; (**D**) style and connective, ventral view; (**E**) aedeagus, posterior view; (**F**) aedeagus, lateral view.

Figure 8A–C, Figure 9A–F, Figure 24G, and Figure 25G1–G3.

**Description.** Length: ♂ 3.5–3.8 mm, ♀ 3.9–4.0 mm. General color yellowish with brown cloudy markings. Crown relatively blunt and covered with several obscure dark spots between eyes. Vertex with a brownish irregular line extending from near apex to eye, curving around ocelli. Face cream-colored and immaculate. Pronotum yellowish mottled with brown irregular cloudy markings. Scutellum mottled with two large diverging brown patches near basal angles, apical area lighter, with a crescent-like marking, neighboring two distinct dumbbell-shaped pale speckles. Forewings shiny yellow and hyaline, with brown cloudy markings along costal margin and apical portion especially, veins with or without white segments. Ventral surface and legs uniform dull yellow. Female color pattern as in male, but ground-color of crown and vertex cream-colored, which makes the color pattern more distinct.

**Male genitalia.** Pygofer in lateral view longer than its height, with many macrosetae on posterior half, caudal margin dentate; internal process tooth-like, directed ventrad. Valve short and rectangular. Subgenital plate extending posteriorly farther than pygofer apex, narrow, triangular, gradually tapered in apical 1/2, with a row of macrosetae in middle and several microsetae along inner margins. Style slender, S-shaped, apical dilation of apophysis elongate boot-shaped, strongly curved, without teeth, apex acuminate. Connective cross-shaped, the side arms folded downward. Aedeagus with dorsal apodeme short, shaft moderately long, sinuate, gradually tapered to apex, with pair of slender processes arising apically and extended basolaterad, 1/3 length of shaft. Gonopore ventral, situated near basal 1/3 of shaft.

**Female.** Sternite VII posterior margin truncate with acute median notch. Second valvulae with preapical angle 90°. Third valvula dorsal margin shallowly concave preapically.

**Material examined. Holotype** ♂, THAILAND: Chiang Mai, Doi Phahompok NP Kewlom1/montane forest, 20°3.549′ N, 99°8.552′ E, 2174 m, Malaise trap, 7–14.ii.2008, Seesom. K. (QSBG). Paratypes: 3♂♂3♀♀, same data as holotype (INHS); 1♂, Chiang Mai, Doi Phahompok NP Doi Phaluang, 20°1.06′ N 99°9.581′ E, 1449 m, Malaise trap, 7–14.ii.2008, Seesom.K. (NWAFU).

**Distribution.** Thailand (Chiang Mai) (Figure 27).

**Etymology.** The species name is a Latin adjective, which refers to the aedeagal shaft with two short processes.

**Remarks.** This species can be identified by the aedeagus with a single pair of apical processes; all other species with two pairs of processes or no process arising apically or subapically.

#### 3.3.7. *Xestocephalus densprint* sp. nov.

**Figure 10:**
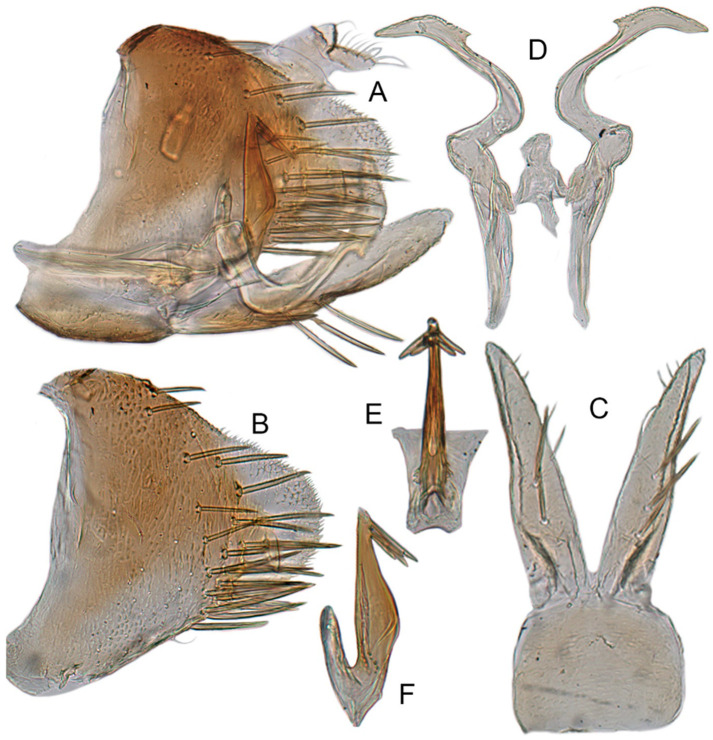
*Xestocephalus densprint* sp. nov. (**A**) Male genital capsule, lateral view; (**B**) pygofer lobe, lateral view; (**C**) subgenital plates and valve, ventral view; (**D**) style and connective, ventral view; (**E**) aedeagus, posterior view; (**F**) aedeagus, lateral view.

Figure 8D–F, Figure 10A–F, Figure 24H, and Figure 25H1–H3.

**Description.** Length: ♂ 3.6–3.8 mm, ♀ 3.9–4.1 mm. General color yellowish with brown cloudy markings. Crown relatively pointed and covered with several obscure dark spots between eyes. Vertex with a light brown irregular line extending from near apex to eye, curving around ocelli. Face cream-colored and immaculate. Pronotum yellowish mottled with light brown irregular cloudy markings. Basal triangles of scutellum dull orange with brown crescent-like margins. Forewings shiny yellow and hyaline, with brown cloudy markings along costal margin and apical portion especially, veins with or without intermittent white segments. Ventral surface and legs uniform dull yellow. Female color slightly darker than male.

**Male genitalia.** Pygofer in lateral view longer than height, lobe with approximately 22 macrosetae on posterior half, with caudal margin dentate. Internal process of pygofer elongate, thornlike, directed ventrad. Valve short and rectangular. Subgenital plate narrow, triangular, gradually tapered in apical 1/2, with 2 or 3 macrosetae in middle and several microsetae along inner margins. Style slender, S-shaped, apical dilation of apophysis elongate boot-shaped, strongly curved and tapered gradually, without distinct teeth. Connective cross-shaped, side arms folded downward. Aedeagus with shaft much longer than apodeme, anterior margin straight and posterior margin protruded at middle, with a row of basal denticles on each side and two pairs of slender apical processes directed lateroventrally, upper processes longer than lower ones. Gonopore ventral, situated near basal 1/3 of shaft.

**Female.** Sternite VII posterior margin truncate with narrow acute median notch. Second valvulae with ventral preapical angle 90°. Third valvula dorsal margin very shallowly concave preapically.

**Material examined. Holotype** ♂, THAILAND: Chiang Mai, Doi Inthanon NP Checkpoint 2, 18°31.554′ N, 98°29.94′ E, 1700 m, Malaise trap, 9–16.ii.2007, Y. Areeluck (QSBG); Paratypes: 8♂♂3♀♀, same data as holotype (INHS); 1♂1♀, Chiang Mai, Doi Inthanon NP Checkpoint 2, 18°31.554′ N, 98°29.94′ E, 1700 m, Malaise trap, 16–23.iii.2007, Y. Areeluck (NWAFU).

**Distribution.** Thailand (Chiang Mai) (Figure 27).

**Etymology.** The species name is the Latin word “densprint”, which refers to the base of the aedeagal shaft with two rows of odontoid processes.

**Remarks.** The new species is similar to *X. binatus* Cai and He [84] in aedeagal structure but distinguished by the aedeagal shaft with apical processes shorter than 2/5 length of shaft, with a row of basal denticles on each side (aedeagus with apical processes equal to 1/2 length of shaft, without a vrow of basal denticles on each side in *X. binatus*).

#### 3.3.8. *Xestocephalus toroensis* Matsumura, 1914, n. rec.

**Figure 11:**
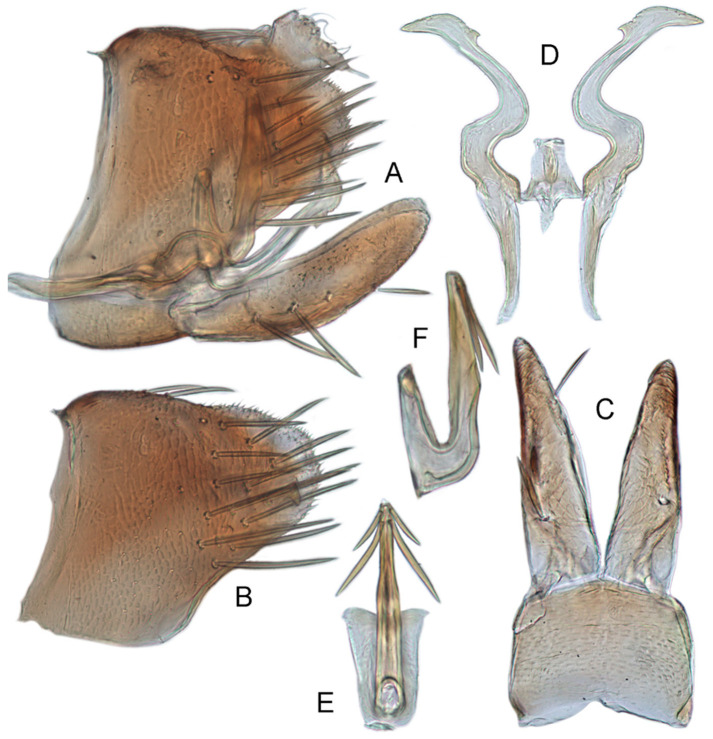
*Xestocephalus toroensis* Matsumura, 1914, n. rec. (**A**) Male genital capsule, lateral view; (**B**) pygofer lobe, lateral view; (**C**) subgenital plates and valve, ventral view; (**D**) style and connective, ventral view; (**E**) aedeagus, posterior view; (**F**) aedeagus, lateral view.

Figure 8G–I, Figure 11A–F, Figure 24I, and Figure 25I1–I3.

*Xestocephalus toroensis* Matsumura, 1914: 199 [59]; Kamitani, 2005: 42 Figures 13–18 [32].

**Redescription.** Length: ♂ 2.7–2.9 mm, ♀ 3.7–3.9 mm. General color yellowish with brown cloudy markings. Crown covered with two obscure semicircular brown markings between eyes. Vertex with intermittent brownish line extending from near apex to eye, curving around ocelli. Face yellow and immaculate. Pronotum yellowish mottled with obscure brown cloudy markings, especially along lateral margins. Scutellum yellow, mottled with two diverging dark patches near basal angles, apical area light, with a crescent-like marking. Forewings yellow and hyaline, with brown cloudy markings along costal margin and apical portion especially, shadowed along veins. Ventral surface and legs uniform yellow.

**Male genitalia.** Pygofer in lateral view as long as high, gradually narrowed to round caudal margin, with many macrosetae on posterior half; internal process tooth-like and directed ventrad. Valve short and trapezoidal. Subgenital plate narrow, triangular, gradually tapered in apical 1/2, with 2 or 3 macrosetae near middle and several short microsetae along inner margins. Style slender, S-shaped, apical dilation of apophysis relatively broad, boot-shaped, strongly curved and tapering, and basal 1/3 with slight incision on posterior margin, without distinct teeth. Connective cross-shaped, the side arms folded downward. Aedeagus with dorsal apodeme 1/2 as long as shaft; shaft moderately long, anterior margin straight and posterior margin slightly protruded at middle, with two pairs of relatively long, slender apical processes directed lateroventrally, upper processes shorter than lower ones. Gonopore ventral, situated near basal 1/2 of shaft.

**Female.** Sternite VII posterior margin broadly concave with narrow median notch. Second valvulae with ventral preapical angle 90°. Third valvula dorsal margin deeply concave preapically.

**Material examined.** 1♂, THAILAND: Lampang, Chae Son NP Campground#3, 18°49.757′ N, 99°28.266′ E, 487 m, Malaise trap, 1–8.x.2007, Bunruen Kwunnui and Acharaporn Sukpeng (QSBG); 1♂1♀, Nakhon Si, Thammarat Namtok Yong NP Behind campground lavatory, 8°10.434′ N, 99°44.508′ E, 95 m, Malaise trap, 22–29.xii.2008, U-prai.K.; 1♂, Kamphaeng, Phet Mae Wong NP Chong Yen, 16°5.968′ N, 99°6.472′ E, 1306 m, Malaise trap, 1–8.x.2007, Chumpol Piluk and Aram Inpuang (INHS); 2♂♂1♀, Loei, Phu Ruea NP office, 17°28.826′ N, 101°21.33′ E, 860 m, Pan trap, 11–12.vii.2006, Patikhom Tamtip (NWAFU).

**Distribution.** Thailand (Lampang, Nakhon Si, Kamphaeng, Loei) (Figure 27), China (Taiwan), Japan.

**Remarks.***X. toroensis* was described by Matsumura [59] based on three male and one female specimens from Taiwan, China. Kamitani [32] redescribed and illustrated the genitalia for the first time. It resembles *X. binatus* Cai and He [84] but differs in having the upper processes of the aedeagus shorter than the lower ones (upper processes longer than lower ones in *X. binatus*).

#### 3.3.9. *Xestocephalus chrysanthemum* sp. nov.

**Figure 12:**
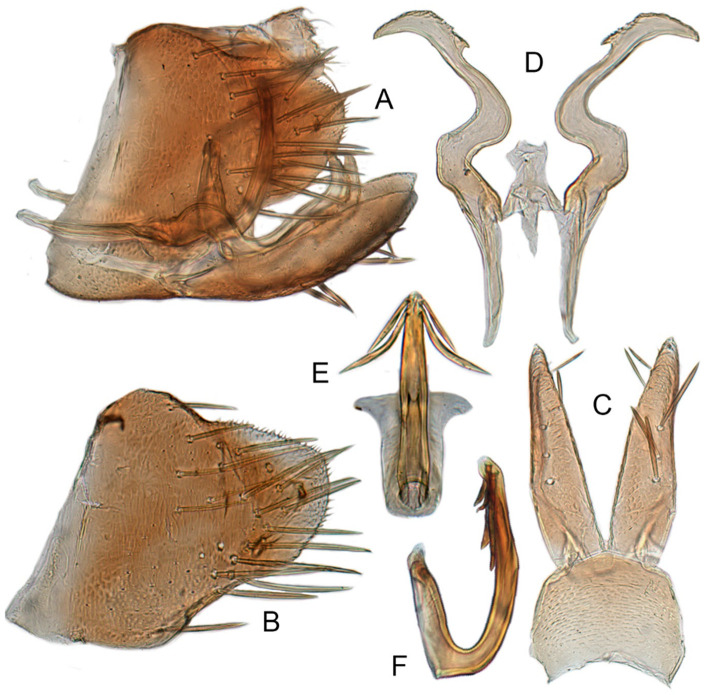
*Xestocephalus chrysanthemum* sp. nov. (**A**) Male genital capsule, lateral view; (**B**) pygofer lobe, lateral view; (**C**) subgenital plates and valve, ventral view; (**D**) style and connective, ventral view; (**E**) aedeagus, posterior view; (**F**) aedeagus, lateral view.

Figure 8J–L, Figure 12A–F, Figure 24J, and Figure 26A1–A3.

**Description.** Length: ♂ 2.9–3.0 mm, ♀ 3.1 mm. General color yellowish with brown cloudy markings. Crown covered with two semicircular brown markings between eyes. Vertex with intermittent brownish line extending from near apex to eye, curving around ocelli. Face cream-colored and immaculate. Pronotum yellowish mottled with dense brown cloudy markings especially along lateral margins. Each angle of scutellum mottled with crescent-like marking and shadow. Forewings shiny yellow and hyaline, with brown cloudy markings along costal margin and apical portion especially along veins. Ventral surface and legs uniform faint yellow.

**Male genitalia.** Pygofer in lateral view as long as high, gradually narrowed to round posterior margin, with many macrosetae on posterior half; internal process tiny, tooth-like, directed ventrad. Valve short and trapezoid. Subgenital plate narrow, triangular, gradually tapered in apical 1/2, with 3 macrosetae in middle and several short microsetae along inner margins. Style slender, S-shaped, apical dilation of apophysis large, boot-shaped, strongly curved, tapered, basal 1/3 of posterior margin with slight incision, apical margin irregular but without distinct teeth. Connective cross-shaped, the side arms folded downward. Aedeagus with dorsal apodeme 1/2 as long as shaft; shaft moderately long, curved dorsad and tapering gradually, with two pairs of apical processes directed lateroventrally, upper processes 1/2 as long as lower ones and curved anterad. Gonopore ventral, situated near basal 1/2 of shaft.

**Female.** Sternite VII posterior margin shallowly concave with narrow median notch. Second valvulae with ventral preapical angle 90°. Third valvula dorsal margin very shallowly concave.

**Material examined. Holotype** ♂, THAILAND: Nakhon Si, Thammarat Namtok Yong NP Road to Khao Mhen, 8°16.959′ N 99°39.149′ E, 499 m, Malaise trap, 20–27.viii.2008, Samnaokan. S (QSBG). Paratypes, 1♂, Kamphaeng, Phet Mae Wong NP Chong Yen, 16°5.212′ N 99°6.576′ E, 1306 m, Malaise trap, 8–15.x.2007, Piluek C. and Inpuang A. (QSBG); 1♂, Chiang Mai, Doi Chiang Dao WS Nature Trail, 19°24.187′ N 98°55.312′ E, 491 m, Malaise trap, 28.viii–4.ix.2007, Songkrant Jagsu and Apichat Watwanich (INHS); 1♂1♀, Chiang Mai, Doi Chiang Dao WS Nature Trail, 19°24.187′ N 98°55.312′ E, 491 m, Malaise trap, 31.vii–7.viii.2007, Songkrant Jagsu and Apichat Watwanich (NWAFU).

**Distribution.** Thailand (Nakhon Si, Kamphaeng, Chiang Mai) (Figure 27).

**Etymology.** The species name “chrysanthemum” is a plant genus name and Latin noun and refers to the processes of the resemblance of the aedeagal shaft to Chrysanthemum petals.

**Remarks.** The new species is similar to *X. toroensis* Matsumura [32] but differs in having the aedeagal shaft distinctly curved with the upper pair of distal processes curved anterad (aedeagal shaft slightly curved with two pairs of distal processes straight in *X. toroensis*).

#### 3.3.10. *Xestocephalus tenusis* sp. nov.

**Figure 13:**
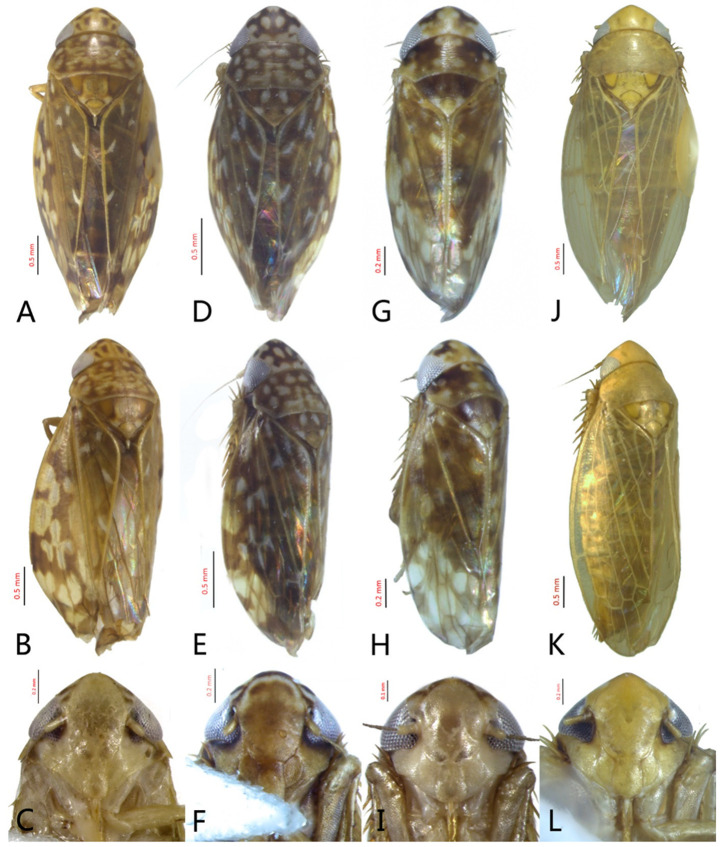
Male habitus of *Xestocephalus*. (**A**–**C**) *X.* *tenusis* sp. nov.; (**D**–**F**) *X.* *exproiecturus* sp. nov.; (**G**–**I**) *X. abyssinicus*; (**J**–**L**) *X.* *cowboyocreus* sp. nov. (**A**,**D**,**G**,**J**) dorsal view; (**B**,**E**,**H**,**K**) latero-dorsal view; (**C**,**F**,**I**,**L**) face.

**Figure 14:**
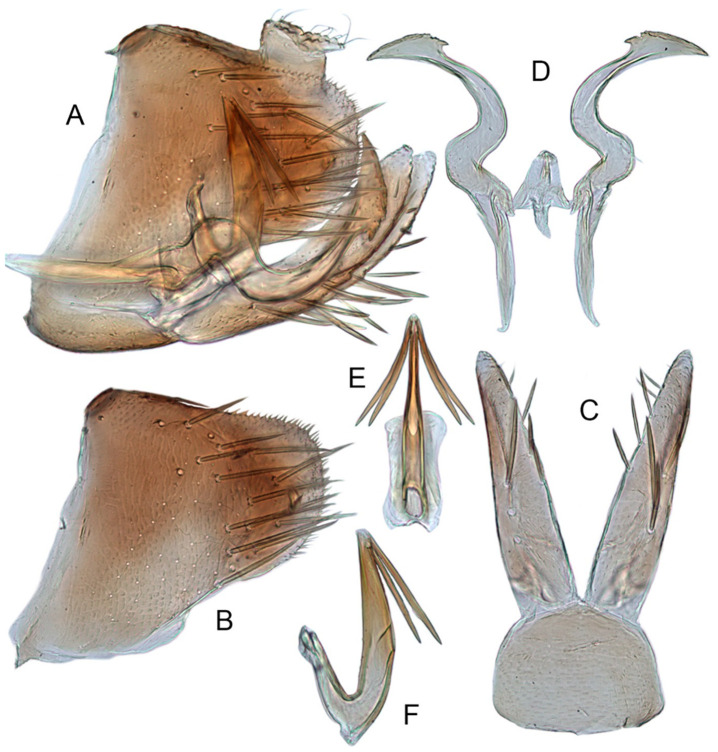
*Xestocephalus tenusis*, sp. nov. (**A**) Male genital capsule, lateral view; (**B**) pygofer lobe; (**C**) subgenital plates and valve, ventral view; (**D**) style and connective, ventral view; (**E**) aedeagus, posterior view; (**F**) aedeagus, lateral view.

Figure 13A–C, Figure 14A–F, Figure 24K, and Figure 26B1–B3.

**Description.** Length: ♂ 3.6–3.7 mm, ♀ 3.8–4.0 mm. Yellowish with brown cloudy markings. Crown relatively pointed and covered with several obscure dark spots between eyes. Vertex with brownish irregular line extending from near apex to eye, curving around ocelli. Face yellowish and immaculate. Pronotum yellowish mottled with brown irregular cloudy markings especially along lateral margins. Scutellum mottled with brown irregular cloudy markings near basal angles, apical area lighter, with a crescent-like marking. Forewings shiny yellow and hyaline, with brown cloudy markings along costal margin and apical portion especially, shadowed along veins. Ventral surface and legs uniform yellowish.

**Male genitalia.** Pygofer in lateral view longer than high, with approximately 19 macrosetae on posterior half; internal process triangular, directed ventrad. Valve short and trapezoidal. Subgenital plate narrow, triangular, gradually tapered in apical 1/2, furnished with 3 macrosetae in apical 2/3 and several short macrosetae along inner margins. Style slender, S-shaped, apical dilation of apophysis broad, boot-shaped, evenly curved, tapered gradually, basal 1/3 of distal margin with slight incision, without distinct teeth. Connective cross-shaped, the side arms folded downward. Aedeagus with dorsal apodeme 1/2 as long as shaft; shaft moderately long, slightly curved anteriorly and broadened medially, tapering to blunt apex in lateral and ventral aspect, with two pairs of long, slender, nearly straight apical processes directed ventroposteriorly, upper processes as long as lower ones. Gonopore ventral, situated near midlength of shaft.

**Female.** Sternite VII posterior margin shallowly concave with narrow median notch. Second valvulae ventral preapical angle 90°. Third valvula dorsal margin deeply concave preapically.

**Material examined. Holotype** ♂, THAILAND: Chiang Mai, Doi Inthanon NP Checkpoint 2, 18°31.554′ N, 98°29.94′ E, 1700 m, Malaise trap, 1–8.v.2007, Y. Areeluck (QSBG); Paratypes, 1♂3♀♀, data same as holotype (INHS); 1♂, Chaiyaphum, Pa Hin Ngam NP Nature trail at Lan Hin Nau, 15°37.615′ N, 101°23.436′ E, 668 m, Malaise trap, 19–26.ix.2006, Katae Sa-nog and Buakaw Adnafai (NWAFU).

**Distribution.** Thailand (Chiang Mai, Chaiyaphum) (Figure 27).

**Etymology.** The species name is a Latin word meaning slender, which refers to the two pairs of slender processes of the aedeagal shaft.

**Remarks.** This species resembles *X. binatus* Cai and He [84] but can be identified by the two pairs of equally long, straight, and slender apical processes on the aedeagal shaft (upper processes shorter than lower ones in *X. binatus*).

#### 3.3.11. *Xestocephalus exproiecturus* sp. nov.

**Figure 15:**
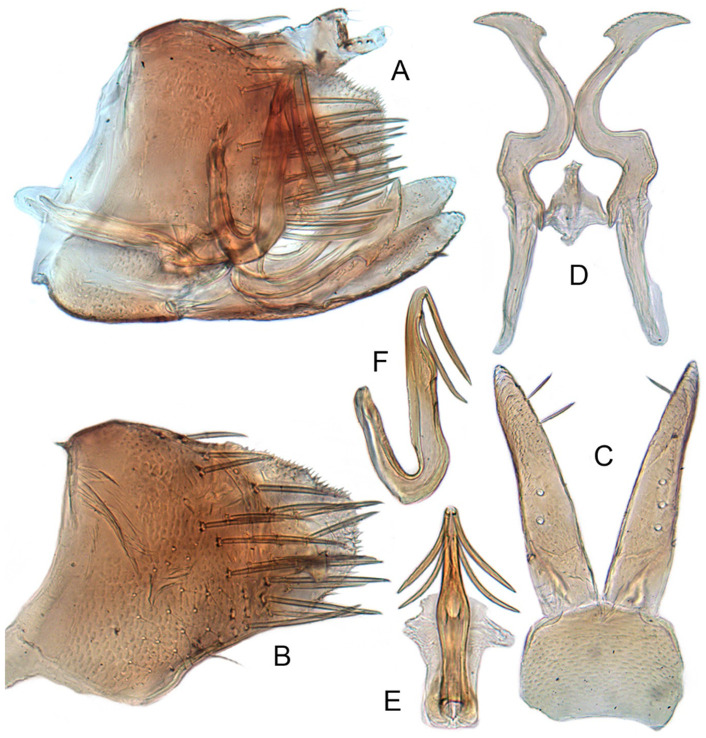
*Xestocephalus exproiecturus* sp. nov. (**A**) Male genital capsule, lateral view; (**B**) pygofer lobe; (**C**) subgenital plates and valve, ventral view; (**D**) style and connective, ventral view; (**E**) aedeagus, posterior view; (**F**) aedeagus, lateral view.

**Figure 16:**
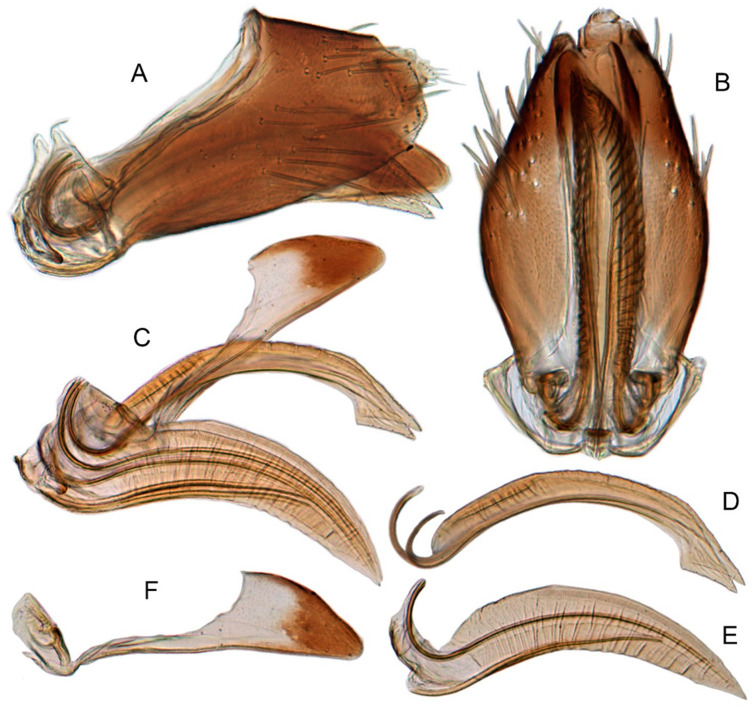
*Xestocephalus exproiecturus* sp. nov. (**A**) Female pygofer, lateral view; (**B**) female pygofer, ventral view; (**C**) ovipositor of female, lateral view; (**D**) second valvulae of female ovipositor, lateral view; (**E**) first valvula of female ovipositor, lateral view; (**F**) third valvula of female ovipositor, lateral view.

Figure 13D–F, Figure 15A–F, Figure 16A–F, Figure 24L, and Figure 26C1–C3.

**Description.** Length: ♂ 2.8–3.0 mm, ♀ 3.1–3.2 mm. General color brownish with numerous cream-colored spots. Crown covered with four cream-colored spots and posterior margin cream-colored between eyes. Vertex with three small whitish spots, ocelli situated in white dots on each side; each side with short whitish stripe connecting ocellus and eye. Face light brown and immaculate. Pronotum brown mottled with evenly distributed cream-colored spots. Scutellum brown, with a crescent-like marking flanked by four distinct pale speckles. Forewings shiny yellow and hyaline, with brown cloudy markings along costal margin and apical portion especially, veins intermittently white and brown. Ventral surface and legs uniform pale brown.

**Male genitalia.** Pygofer in lateral view longer than high, gradually narrowed to round caudal margin, with many macrosetae on posterior half; internal process tooth-like and directed ventrad. Valve short and trapezoidal. Subgenital plate triangular, broadened at base and narrowing to apex, with 2–3 macrosetae near middle and several microsetae along inner margin. Style slender, S-shaped, apical dilation of apophysis relatively short and broad, boot-shaped, apical margin evenly convex, without teeth, apex evenly tapered. Connective cross-shaped, the side arms folded downward. Aedeagus with dorsal apodeme slightly longer than half shaft length, shaft straight. Posterior margin irregularly curved in lateral view, abruptly narrowed at apical 1/3, with paired long, straight apical and subapical processes directed lateroventrally, apical processes as long as subapical ones. Gonopore ventral, situated near midlength of shaft.

**Female.** Sternite VII posterior margin slightly convex with slight median notch. Second valvulae preapical ventral angle obtuse. Third valvular dorsal margin deeply concave.

**Material examined. Holotype** ♂, THAILAND: Phetchabun, Thung Salaeng Luang NP Gang wang nam yen 3, 16°34.36′ N, 100°52.83′ E, 725 m, Malaise trap, 23–30.vii.2007, Pongpitak and Sathit (QSBG). Paratypes: 1♂, data same as holotype (QSBG); 1♂, THAILAND: Phetchabun, Nam Nao NP Pine forest/Sambon 1, 16°42.47′ N, 101°35.26′ E, 872 m, Malaise trap, 16–23.x.2006, Noopean Hongyothi; 1♂, Phetchabun, Nam Nao NP Pine forest/Sambon 3, 16°42.41′ N, 101°35.3′ E, 868 m, Malaise trap, 16–23.x.2006, Noopean Hongyothi; 1♂, Phetchabun, Nam Nao NP Pine forest/Sambon 3, 16°42.41′ N, 101°35.3′ E, 868 m, Malaise trap, 2–9.x.2006, Leng Janteab; 1♂, Phetchabun, Thung Salaeng Luang NP Pine forest Gang wang nam yen, 16°35.789′ N, 100°52.286′ E, 769 m, Malaise trap, 22–28.xii.2006, Pongpitak and Sathit; 1♂2♀♀, Phetchabun, Thung Salaeng Luang NP Pine forest Gang wang nam yen, 16°35.789′ N, 100°52.286′ E, 769 m, Malaise trap, 4–11.ix. 2007, Pongpitak and Sathit; 2♂♂2♀♀, Phetchabun, Nam Nao NP Pine forest/Sambon 3, 16°42.41′ N, 101°35.3′ E, 868 m, Malaise trap, 25.ix–2.x.2006, Leng Janteab; 2♂♂, Phetchabun, Thung Salaeng Luang NP Gang wang nam yen 2, 16°34.34′ N, 100°53.43′ E, 758 m, Malaise trap, 30.vii–6.viii.2007, Pongpitak and Sathit; 2♂♂1♀, Phetchabun, Thung Salaeng Luang NP Gang wang nam yen 1, 16°34.57′ N, 100°53.16′ E, 750 m, Malaise trap, 6–13.viii.2007, Pongpitak and Sathit; 1♂, Phetchabun, Thung Salaeng Luang NP Gang Wang Nam Yen, 16°36.587′ N, 100°53.395′ E, 753 m, Malaise trap, 7–14.vi.2007, Pongpitak Pranee and Sathit; 2♂♂, Nakhon Nayok, Khao Yai NP near Training Center 2, 14°24.515′ N, 101°22.432′ E, 750 m, Malaise trap, 19–26.ii.2007, Wirat Sukho; 1♂1♀, Lampang, Chae Son NP Campground#3, 18°49.757′ N, 99°28.266′ E 487 m, Malaise trap, 1–8.x.2007, Bunruen Kwunnui and Acharaporn Sukpeng; 2♂♂, Chanthaburi, Khao Khitchakut NP 500m NW/Prabaht Unit, 12°48.95′ N, 102°9.14′ E, 12 m, Malaise trap, 1–8.ix.2008, Suthida and Charoenchai; 1♂, Chanthaburi, Khao Khitchakut NP 100 m N/Prabaht Unit, 12°48.842′ N, 102°9.144′ E, 203 m, Malaise trap, 28.vii–3.viii.2008, Suthida and Charoenchai (INHS); 1♂, Chanthaburi, Khao Khitchakut NP 50m NE/Prabaht Unit, 12°48.779′ N, 102°9.181′ E, 107 m, Malaise trap, 28.vii–3.viii.2008, Suthida and Charoenchai; 7♂♂3♀♀, Chanthaburi, Khao Khitchakut NP 500m N/Prabaht Unit, 12°48.98′ N, 102°9.14′ E, 152 m, Malaise trap, 15–22.ix.2008, Suthida and Charoenchai; 3♂♂1♀, Chanthaburi, Khao Khitchakut NP 100m N/Prabaht Unit, 12°48.842′ N, 102°9.144′ E, 203 m, Malaise trap, 21–28.vii.2008, Charoenchai and Suthida; 1♂, Chanthaburi, Khao Khitchakut NP Campground/Prabaht Unit, 12°48.852′ N, 102°9.204′ E, 99 m, Malaise trap, 28.vii–3.viii.2008, Suthida and Charoenchai (NWAFU).

**Distribution.** Thailand (Phetchabun, Nakhon Nayok, Lampang, Chanthaburi) (Figure 27).

**Etymology.** The species name combines the Latin words “ex” + “proiecturus”, which refers to the separation of the base of two pairs of processes from the aedeagal shaft.

**Remarks.** The new species is similar to *X. tenusis* sp. nov. but differs in having the aedeagal shaft abruptly narrowed at the apical 1/3 in lateral view and in having the bases of the two pairs of shaft processes distinctly separated (aedeagus slightly curved anteriorly and broadened medially, tapering to blunt apex, with bases of two pairs of shaft processes distinctly close-set in *X. tenusis*).

#### 3.3.12. *Xestocephalus abyssinicus* Heller and Linnavuori, 1968, n. rec.

**Figure 17:**
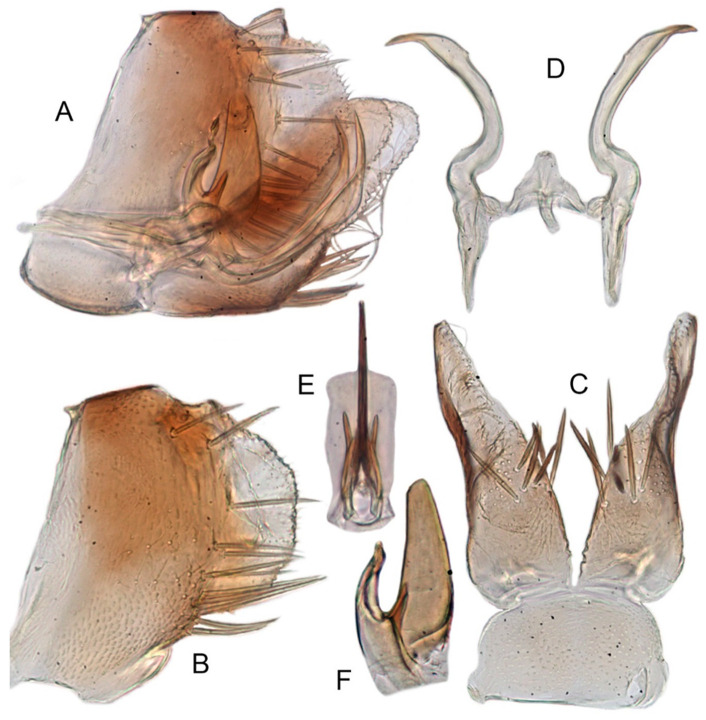
*Xestocephalus abyssinicus* Heller and Linnavuori, 1968, n. rec. (**A**) Male genital capsule, lateral view; (**B**) pygofer lobe, lateral view; (**C**) subgenital plates and valve, ventral view; (**D**) style and connective, ventral view; (**E**) aedeagus, posterior view; (**F**) aedeagus, lateral view.

Figure 13G–I, Figure 17A–F, Figure 24M, and Figure 26D1–D3.

*Xestocephalus abyssinicus* Heller and Linnavuori, 1968:14, Figures 7–10 [85].

**Description.** Length: ♂ 2.3–2.5 mm, ♀ 2.5–2.7 mm. General color yellowish with numerous pale patches and brown cloudy markings. Crown relatively blunt, covered with several pale obscure spots between eyes. Vertex with a brownish irregular line extending from near apex to eye, curving around ocelli. Face cream-color, immaculate extending to vertex. Pronotum brown, two elongated pale spots in middle and two round pale spots next to lateral margin, respectively. Scutellum brown, apical area lighter with a crescent-like marking. Forewings shiny yellow, with many elongate hyaline patches and brown cloudy markings all over; veins surrounded with shadows. Ventral surface and legs uniform light yellow.

**Male genitalia.** Pygofer in lateral view higher than long, furnished with approximately 15–20 macrosetae on posterior half, caudal margin dentate and several distinct notches along ventral margin posterior margin. Internal process of pygofer tooth-like and directed ventrad. Valve short and trapezoidal. Subgenital plate broad, linguiform, apical portion rounded, lateral margins of apex 2/3 slightly curved inwards, with group of macrosetae near base and several rows of hairlike setae at elevation near inner margin. Style slender, S-shaped, subapical dilation of apophysis indistinct, preapical heel poorly developed, apex tapered, without teeth. Connective cross-shaped, the side arms folded downward. Aedeagus symmetrical basal apodeme shorter and much slender than shaft in lateral view, shaft strongly compressed; atrium broad, with pair of short, slender processes extended dorsad from junction of shaft and atrium; shaft narrowed to obliquely truncate apex in lateral aspect, very slender in posterior aspect. Gonopore slit-like near basal 1/3 of shaft.

**Female.** Sternite VII truncate with slight medial notch. Second valvulae with preapical ventral angle 90°. Third valvula dorsal margin deeply concave preapically.

**Material examined.** 4♂♂3♀♀, THAILAND: Prachuab, Khiri Khan Khao Sam Roi Yot NP Laem Sala beach, 12°12.234′ N, 100°0.767′ E, Malaise trap, 20–27.vii.2008, Yai and Amnad (QSBG); 5♂♂5♀♀, Prachuab, Khiri Khan Khao Sam Roi Yot NP Nursery, 12°7.58′ N, 99°57.478′ E, Malaise trap, 13–20.vii.2008, Amnad and Yai (3♂♂3♀♀ in INHS, 2♂♂2♀♀ in NWAFU).

**Distribution.** Thailand (Prachuab) (Figure 27), Ethiopia.

**Remarks.***X. abyssinicus* was described by Heller and Linnavuori [83] based on a male specimen from Ethiopia. This is the first record of the species from Asia. It is similar to *X. nonattribus* in the shape of aedeagus and styles but distinguished by the presence of a pair of basal processes at the junction of the aedeagal shaft and atrium. The aedeagal processes of the specimens from Thailand are longer than those illustrated for the holotype by Heller and Linnavuori [83] but other aspects of the male genitalia, including the strongly compressed aedeagal shaft and distinctively shaped style apex, are nearly identical, so we consider the observed variation to be intraspecific.

#### 3.3.13. *Xestocephalus cowboyocreus* sp. nov.

**Figure 18:**
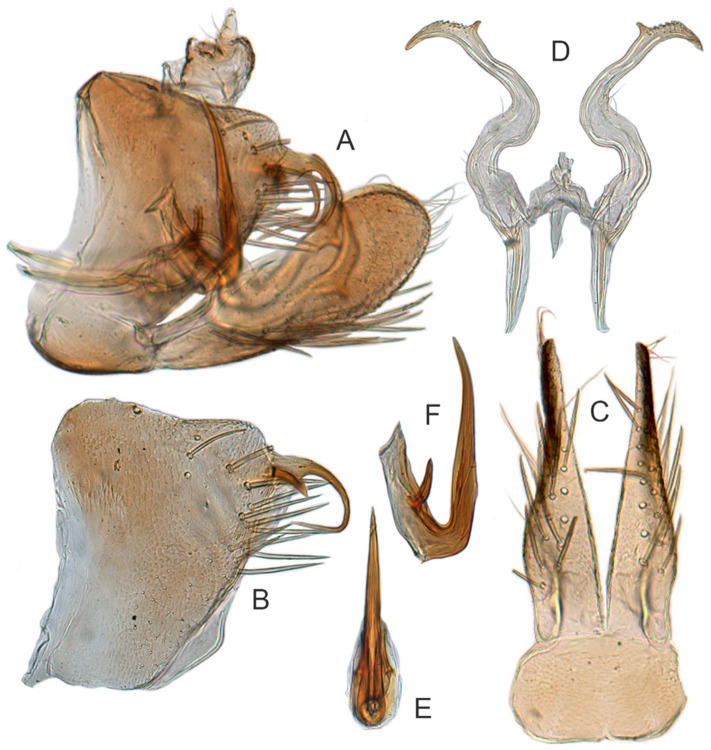
*Xestocephalus cowboyocreus* sp. nov. (**A**) Male genital capsule, lateral view; (**B**) pygofer lobe, lateral view; (**C**) subgenital plates and valve, ventral view; (**D**) style and connective, ventral view; (**E**) aedeagus, posterior view; (**F**) aedeagus, lateral view.

Figure 13J–L, Figure 18A–F, Figure 24N, and Figure 26E1–E3.

**Description.** Length: ♂ 5.0–5.3 mm, ♀ 5.1–5.4 mm. Body nearly uniformly yellowish and immaculate. Crown, vertex, face, and pronotum yellowish except area around ocelli whitish. Apex of crown pointed and distinctly produced. Angles of scutellum mottled with crescent-like markings. Forewings shiny yellow, hyaline and immaculate. Ventral surface and legs uniform yellow.

**Male genitalia.** Pygofer in lateral view higher than long, with many macrosetae on posterior margin, with posterodorsal process long, curved ventrad then recurved anteriorly; internal process dagger-like and directed ventrad. Valve short and rectangular. Subgenital plate extending posteriorly farther than pygofer apex, broad, linguiform, basal half horizontal and apical half vertical, with apices broadly rounded in lateral view, with multiseriate macrosetae and serially arranged lateral microsetae. Style S-shaped, apical dilation of apophysis scythe-shaped with apical 1/2 tapered strongly, outer margin dentate and provided with prominent spine. Connective cross-shaped, the side arms folded downward. Aedeagal shaft slender and tapering gradually to apex, subapex slightly curved anteriorly in lateral aspect, atrium shorter and broader than shaft in lateral view, with pair of short processes on middle projecting dorsad. Gonopore ventral, situated near midlength of shaft.

**Female.** Sternite VII posterior margin truncate with relatively deep median notch. Second valvulae with preapical ventral angle obtuse. Third valvula dorsal margin deeply concave preapically.

**Material examined.** Holotype ♂, THAILAND: Chiang Mai, Doi Inthanon NP Checkpoint 2, 18°31.554′ N, 98°29.94′ E, 1700 m, Malaise trap, 1–8.v.2007, Y. Areeluck (QSBG). Paratypes: 1♂♂3♀♀, same data as holotype (QSBG); 2♂♂1♀, Chiang Mai, Doi Inthanon NP Checkpoint 2, 18°31.554′ N, 98°29.94′ E, 1700 m, Malaise trap, 16–23.iii.2007, Y. Areeluck (INHS); 1♂2♀♀, Chiang Mai, Doi Inthanon NP Checkpoint 2, 18°31.554′ N, 98°29.94′ E, 1700 m, Malaise trap 23.iii–1.v.2007, Y. Areeluck (NWAFU).

**Distribution.** Thailand (Chiang Mai) (Figure 27).

**Etymology.** The species name is a combination of the English word “cowboy” and the Latin “ocreus” (leg), referring to the cowboy-boot-shaped style apex.

**Remarks.** The new species is similar to *X. recipinams* sp. nov. in the structure of the aedeagus but has the aedeagal processes shorter than 1/3 the length of the shaft, the pygofer process long and curved ventrad, and the style apex without a spine on the inner margin (aedeagal processes longer than 1/2 length of shaft, pygofer process curved dorsad and style with 2 or 3 large spines on inner margin in *X. recipinams*).

#### 3.3.14. *Xestocephalus recipinams* sp. nov.

**Figure 19:**
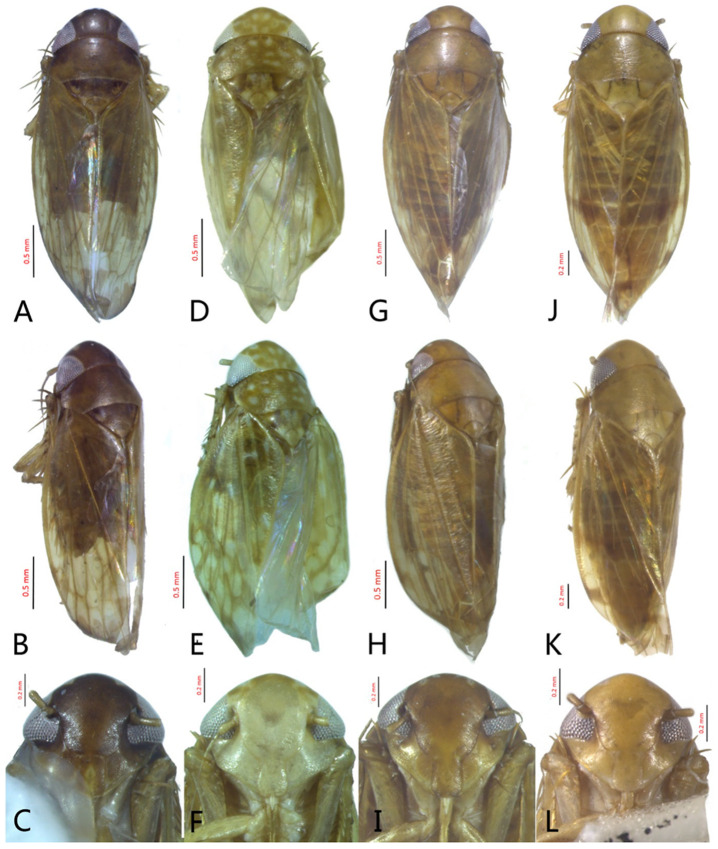
Male habitus of *Xestocephalus*. (**A**–**C**) *X.* *recipinams* sp. nov.; (**D**–**F**) *X.* *limipidissimus* sp. nov.; (**G**–**I**) *X.* *dimiprocessus* sp. nov.; (**J**–**L**) *X.* *malleus*, sp. nov. (**A**,**D**,**G**,**J**) dorsal view; (**B**,**E**,**H**,**K**) laterodorsal view; (**C**,**F**,**I**,**L**) face.

**Figure 20:**
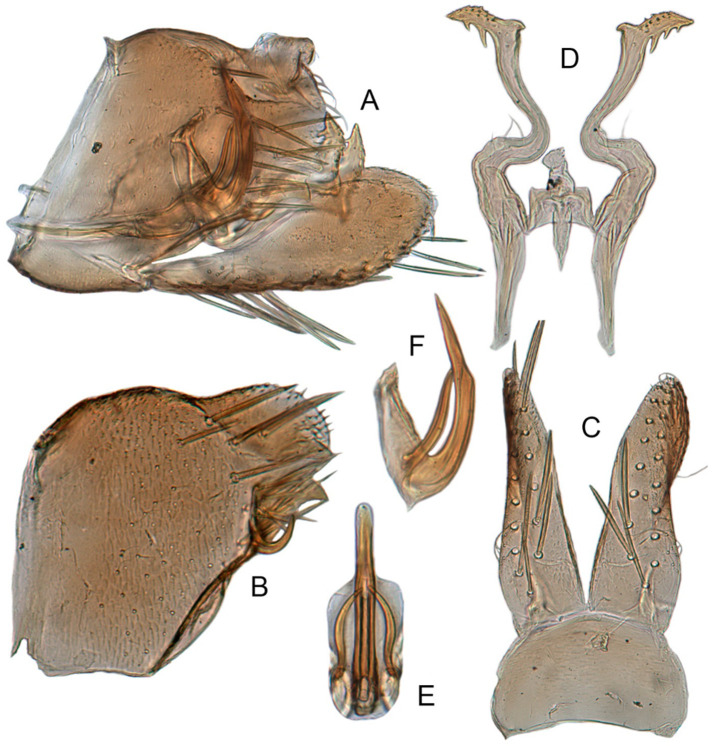
*Xestocephalus recipinams* sp. nov. (**A**) Male genital capsule, lateral view; (**B**) pygofer lobe, lateral view; (**C**) subgenital plates and valve, ventral view; (**D**) style and connective, ventral view; (**E**) aedeagus, posterior view; (**F**) aedeagus, lateral view.

Figure 19A–C, Figure 20A–F, Figure 24O, and Figure 26F1–F3.

**Description.** Length: ♂ 2.9–3.0 mm, ♀ 3.0–3.2 mm. Body light brown and nearly immaculate. Only forewings with brown cloudy markings at apex. Vertex light brown except for ocelli. Face immaculate. Pronotum brown, posterior margin slightly light than anterior margin. Scutellum brown, mottled with two large diverging paler patches near basal angles, apical area light, with a crescent-like marking. Forewings light brown, hyaline, with dark shadows at apex, infuscated along all veins. Ventral surface and legs uniform light brown.

**Male genitalia.** Pygofer in lateral view higher than long, tapered to rounded caudal margin, many macrosetae on posterior half, with two distinct triangular dilations along ventroposterior margin; internal process of pygofer large hook-like and directed dorsad. Valve short and trapezoidal. Subgenital plate broad, linguiform, apex rounded, with two rows of macrosetae. Style S-shaped, apical dilation of apophysis with outer margin concave and provided with scattered teeth; inner margin with 2 or 3 large spines. Connective cross-shaped, the side arms folded downward. Aedeagus with basal apodeme shorter and broader than shaft, with pair of long processes arising near junction with shaft and extended posterodorsad, sinuate in posterior view; shaft slightly curved anteriorly, in lateral view with basal half uniform but apical half tapering to sharp apex. Gonopore ventral, situated near apical 1/3 of the shaft.

**Female.** Sternite VII posterior margin slightly convex with distinct median notch. Second valvulae ventral preapical angle obtuse. Third valvula dorsal margin moderately concave preapically.

**Material examined.** Holotype ♂, THAILAND: Nakhon Si, Thammarat Namtok Yong NP TV aerial, 8°14.262′ N, 99°48.289′ E, 952 m, Malaise trap, 30.iii–6.iv.2009, Paiboon (QSBG). Paratypes: 2♂♂1♀, same data as holotype (INHS); 1♂1♀, THAILAND: Petchaburi, Kaeng Krachan NP Panernthung/km27, 12°49.302′ N, 99°22.263′ E, 950 m, Malaise trap, 18–25.v.2009, Sirichai (NWAFU).

**Distribution.** Thailand (Nakhon Si, Petchaburi) (Figure 27).

**Etymology.** The species name is a Latin word, which refers to the unique appendages of the pygofer.

**Remarks.** The new species is similar to *X. cowboyocreus* sp. nov. in aedeagal structure but differs in having the aedeagal processes more than 1/2 the length of shaft, the pygofer process curved dorsad, and the style with 2 or 3 large spines on the inner margin (aedeagal processes less than 1/3 length of shaft, the pygofer process curved ventrad, and style without spine in *X. cowboyocreus*).

#### 3.3.15. *Xestocephalus limpidissimus* sp. nov.

**Figure 21:**
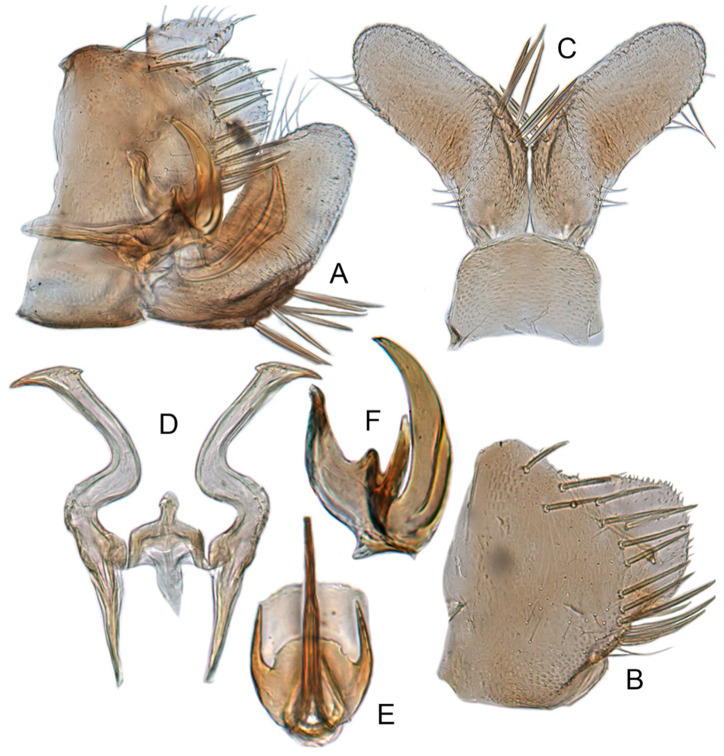
*Xestocephalus limpidissimus* sp. nov. (**A**) Male genital capsule, lateral view; (**B**) pygofer lobe, lateral view; (**C**) subgenital plates and valve, ventral view; (**D**) style and connective, ventral view; (**E**) aedeagus, posterior view; (**F**) aedeagus, lateral view.

Figure 19D–F, Figure 21A–F, Figure 24P, and Figure 26G1–G3.

**Description.** Length: ♂ 2.8 mm, ♀ 3.2 mm. General color dark yellowish with dense cream-colored spots. Crown covered with several irregular pale markings between eyes. Vertex dark yellowish, cream-yellow around ocelli. Face cream-colored and immaculate. Pronotum dark yellowish mottled with even-distributed cream-colored spots. Scutellum mottled with two diverging dark patches at basal angles, apical area light, with a crescent-like marking. Forewings yellow and hyaline, with shadows along costal margin, apical portion, and veins. Ventral surface and legs uniform yellow.

**Male genitalia.** Pygofer in lateral view higher than long, with approximately 17 macrosetae on posterior half; internal processes small and triangular, directed ventrad. Valve short and trapezoidal. Subgenital plate broad, linguiform, apical portion rounded, lateral margins of apical 2/3 slightly curved inwards, with several macrosetae near middle and several rows of hairlike setae near inner margin. Style slender, S-shaped, subapical dilation of apophysis boot-shaped, only slightly curved, without teeth. Connective cross-shaped, the side arms folded downward. Aedeagus with dorsal apodeme slightly shorter than shaft, with a pair of sword-shaped processes on base, directed dorsally, and a short process on middle projecting dorsad; shaft moderately long, compressed, slightly curved anteriorly, tapering to sharply pointed apex in lateral view. Gonopore situated near midlength of shaft.

**Female.** Sternite VII posterior margin broadly concave with distinct median notch. Second valvulae ventral preapical angle obtuse. Third valvula dorsal margin with distinct notch in shallowly concave preapical section.

**Material examined. Holotype** ♂, THAILAND: Lampang, Chae Son NP Campground#3, 18°49.757′ N 99°28.266′ E, 487 m, Malaise trap, 1–8.x. 2007, Bunruen Kwunnui and Acharaporn Sukpeng (QSBG). Paratype: 1♀, same data as holotype (QSBG).

**Distribution.** Thailand (Lampang) (Figure 27).

**Etymology.** The species name is a Latin word meaning lustrous and refers to the crown.

**Remarks.** The new species is similar to *X. japonicus* Ishihara [32] in the shape of the aedeagus in ventral aspect but differs in having the aedeagal shaft thick, the dorsal apodeme with a short process on the middle, and a broader pair of basal processes (aedeagal shaft slender, dorsal apodeme without process on middle, and paired processes slender in *X. japonicus*).

#### 3.3.16. *Xestocephalus dimiprocessus* sp. nov.

**Figure 22:**
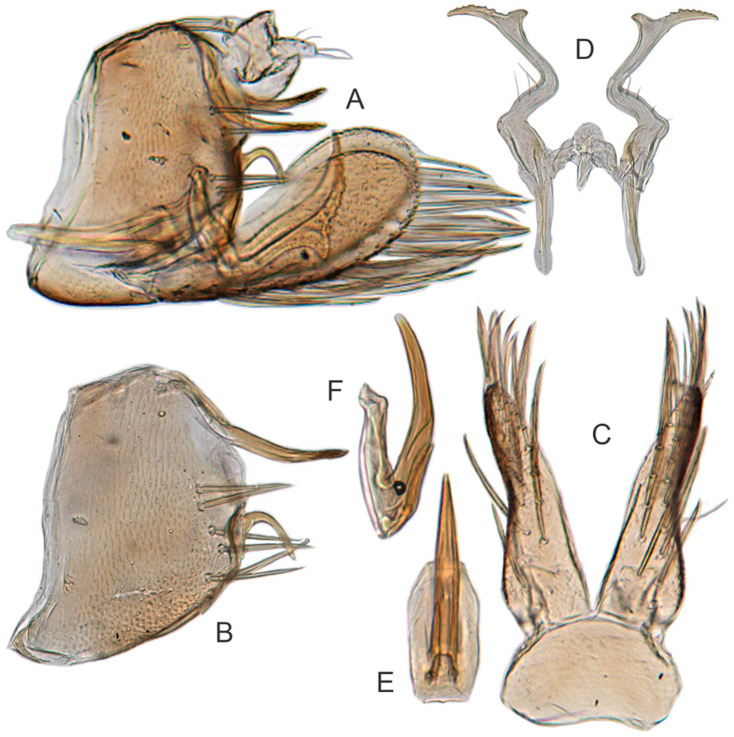
*Xestocephalus dimiprocessus* sp. nov. (**A**) Male genital capsule, lateral view; (**B**) pygofer lobe, lateral view; (**C**) subgenital plates and valve, ventral view; (**D**) style and connective, ventral view; (**E**) aedeagus, posterior view; (**F**) aedeagus, lateral view.

Figure 19G–I, Figure 22A–F, Figure 24Q, and Figure 26H1–H3.

**Description.** Length: ♂ 3.0–3.3 mm, ♀ 3.1–3.4 mm. Body yellowish all over and nearly immaculate. Crown, vertex, face, and pronotum yellowish except around ocelli. Each angle of scutellum mottled with crescent-like marking. Forewings shiny yellow, hyaline, with irregular darker shadows around all veins. Ventral surface and legs uniform dull yellow.

**Male genitalia.** Pygofer relatively short, higher than long, posterodorsal margin with long process directed caudad, with several macrosetae, ventroposterior margin with moderately long process, arched dorsad; internal process large, hook-like and directed ventrad. Valve short and trapezoidal. Subgenital plate extending posteriorly farther than pygofer apex, broad, linguiform, basal half horizontal and apical half vertical, with apex broadly rounded in lateral view, with multiseriate macrosetae and serially arranged lateral microsetae. Style S-shaped, apical dilation of apophysis scythe-shaped, outer margin dentate, with prominent rectangular spine preapically. Connective cross-shaped, the side arms folded downward. Aedeagus with basal apodeme shorter than shaft, shaft slender and slightly curved anteriorly, in lateral view tapered from base to bluntly rounded apex, with pair of basal processes extended along sides of shaft tapering apically. Gonopore ventral, situated near midlength of shaft, lower edge even with apex of processes.

**Female.** Sternite VII posterior margin truncate with distinct median notch. Second valvulae ventral preapical angle obtuse. Third valvula dorsal margin deeply emarginate preapically.

**Material examined.** Holotype ♂, THAILAND: Chiang Mai, Doi Phahompok NP Kewlom1/montane forest, 20°3.549′ N, 99°8.552′ E, 2174 m, Malaise trap, 31.vii–7. viii.2007, Wongchai.P (QSBG). Paratypes: 2♂♂1♀, same data as holotype (INHS); 1♂1♀, Chiang Mai, Doi Phahompok NP Kiewlom1: Montane Forest, 20°3.455′ N, 99°8.551′ E, 2174 m, Malaise trap, 7–14.viii.2007, Komwuan Srisom and Prasit Wongchai (NWAFU).

**Distribution.** Thailand (Chiang Mai) (Figure 27).

**Etymology.** The species name alludes to the overall similarity of this species to *X. biprocessus* but the aedeagal processes are half as long as in the latter.

**Remarks.** The new species is similar to *X. biprocessus* Li and Zhang [35] but can be identified by the pygofer with a long process at the posterodorsal margin, the aedeagal shaft with basal processes less than 1/2 the length of the shaft, and the style apophysis with abprominent rectangular spine preapically (pygofer with short process at posterior margin, aedeagal shaft with basal processes more than 1/2 length of shaft, and style apophysis with triangular spine preapically in *X. biprocessus*).

#### 3.3.17. *Xestocephalus malleus* sp. nov.

**Figure 23:**
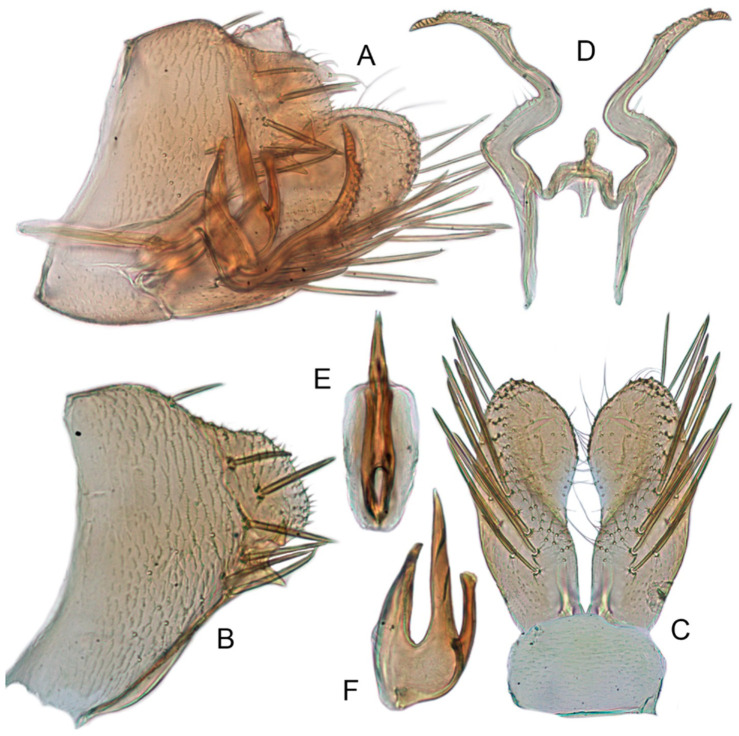
*Xestocephalus malleus* sp. nov. (**A**) Male genital capsule, lateral view; (**B**) pygofer lobe, lateral view; (**C**) subgenital plates and valve, ventral view; (**D**) style and connective, ventral view; (**E**) aedeagus, posterior view; (**F**) aedeagus, lateral view.

**Figure 24:**
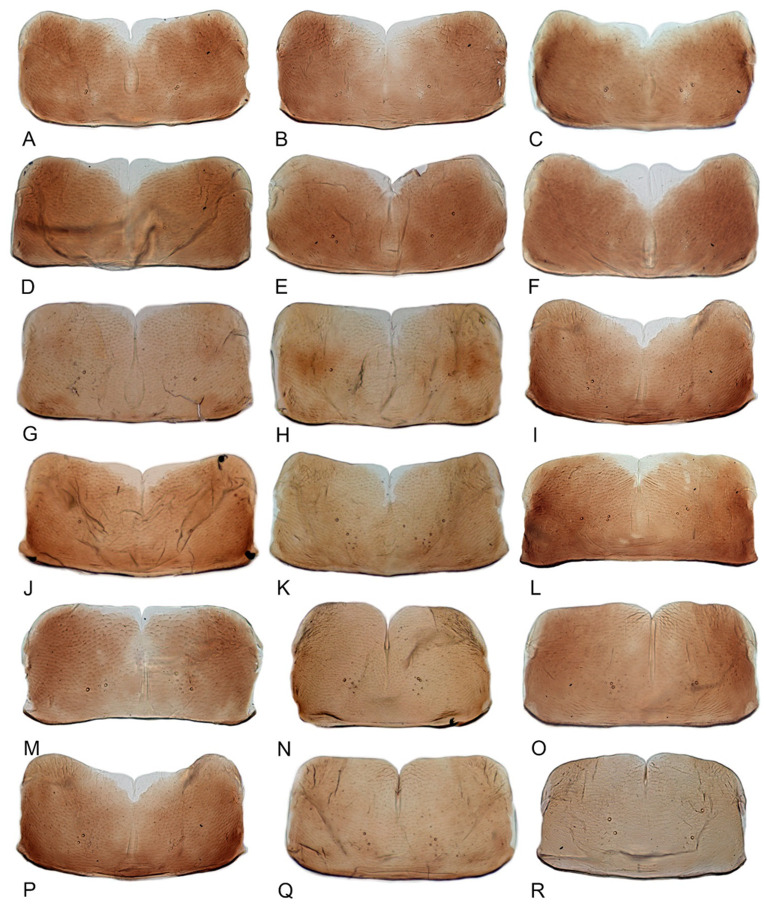
Female sternite VII, ventral view. (**A**) *X. asper*; (**B**) *X. guttulatus*; (**C**) *X. ishidae*; (**D**–**F**) *X. nonattribus* sp. nov.; (**G**) *X. binarius* sp. nov.; (**H**) *X. densprint* sp. nov.; (**I**) *X. toroensis*; (**J**) *X. chrysanthemum* sp. nov.; (**K**) *X. tenusis* sp. nov.; (**L**) *X. exproiecturus* sp. nov.; (**M**) *X. abyssinicus*; (**N**) *X. cowboyocreus* sp. nov.; (**O**) *X. recipinams* sp. nov.; (**P**) *X. limpidissimus* sp. nov.; (**Q**) *X. dimiprocessus* sp. nov.; (**R**) *X. malleus* sp. nov.

**Figure 25:**
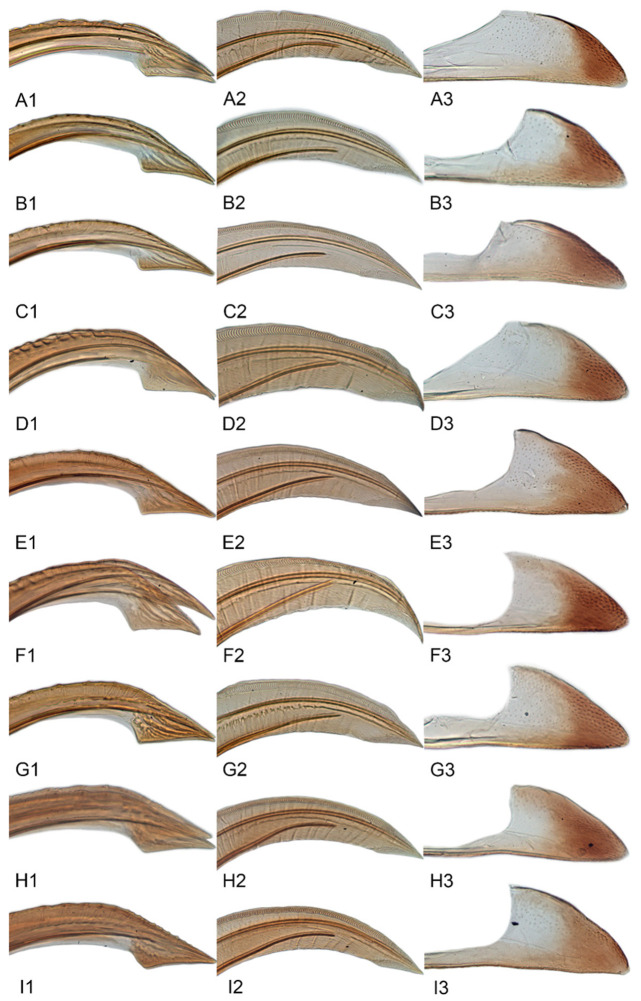
Dissected parts of ovipositor, showing detail of second, first, and third valvulae apex. (**A1**–**A3**) *X. asper*; (**B1**–**B3**) *X. guttulatus*; (**C1**–**C3**) *X. ishidae*; (**D1**–**F3**) *X. nonattribus* sp. nov.; (**G1**–**G3**) *X. binarius* sp. nov.; (**H1**–**H3**) *X. densprint* sp. nov.; (**I1**–**I3**) *X. toroensis*.

**Figure 26:**
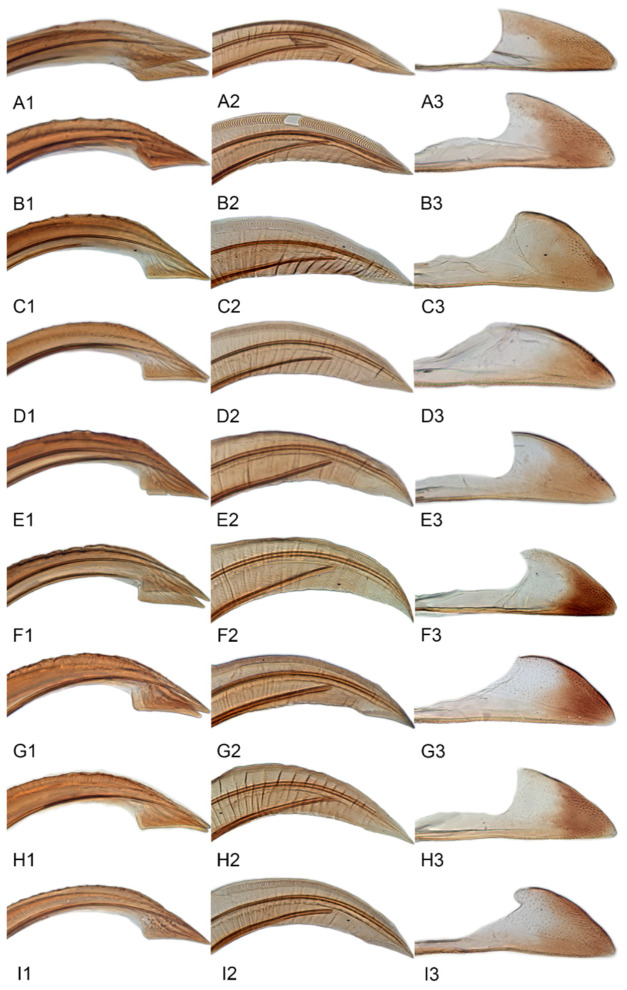
Dissected parts of ovipositor, showing detail of second, first and third valvulae apex. (**A1**–**A3**) *X. chrysanthemum* sp. nov.; (**B1**–**B3**) *X. tenusis* sp. nov.; (**C1**–**C3**) *X. exproiecturus* sp. nov.; (**D1**–**D3**) *X. abyssinicus*; (**E1**–**E3**) *X. cowboyocreus* sp. nov.; (**F1**–**F3**) *X. recipinams* sp. nov.; (**G1**–**G3**) *X. limpidissimus* sp. nov.; (**H1**–**H3**) *X. dimiprocessus* sp. nov.; (**I1**–**I3**) *X. malleus* sp. nov.

Figure 19J–L, Figure 23A–F, Figure 24R, and Figure 26I1–I3.

**Description.** Length: ♂ 2.5–2.6 mm, ♀ 2.7–2.8 mm. Body yellowish all over and immaculate. Crown, vertex, face, and pronotum yellowish except around ocelli whitish. Each angle of scutellum mottled with a crescent-like marking. Forewings shiny yellow and hyaline, only forewings with shadows at apex. Ventral surface and legs uniform yellow.

**Male genitalia.** Pygofer in lateral view higher than long, with many macrosetae on posterior half, caudal margin dentate, with a short spine-like process on ventroposterior corner, directed caudad; internal processes triangular, directed ventrad. Valve short and rectangular. Subgenital plate extending posteriorly farther than pygofer apex, broad, linguiform, basal half horizontal and apical half vertical, with apex broadly rounded in lateral view, with multiseriate macrosetae and serially arranged lateral microsetae. Style S-shaped, apex of apophysis slender, scythe-shaped, outer margin dentate with incision, basal 2/3 of distal part with fish-scale pattern on surface, apical 1/3 narrower with striations. Connective cross-shaped, the side arms folded downward. Aedeagus with basal apodeme shorter than shaft, shaft straight and evenly tapered gradually to a point in lateral aspect, with pair of posterior basal processes parallel to shaft, broadened at apex and directed dorsad. Gonopore ventral near apical 1/3 of shaft.

**Female.** Sternite VII posterior margin slightly convex with distinct median notch. Second valvulae with ventral preapical angle obtuse. Third valvula with dorsal margin deeply concave preapically.

**Material examined. Holotype** ♂, THAILAND: Petchaburi, Kaeng Krachan NP km33/helipad, 12°50.177′ N, 99°20.688′ E, 735 m, Malaise trap, 18–25.v.2009, Sirichai (QSBG). Paratypes: 3♂♂3♀♀, same data as holotype (2♂♂2♀♀ in INHS, 1♂1♀ in NWAFU).

**Figure 27:**
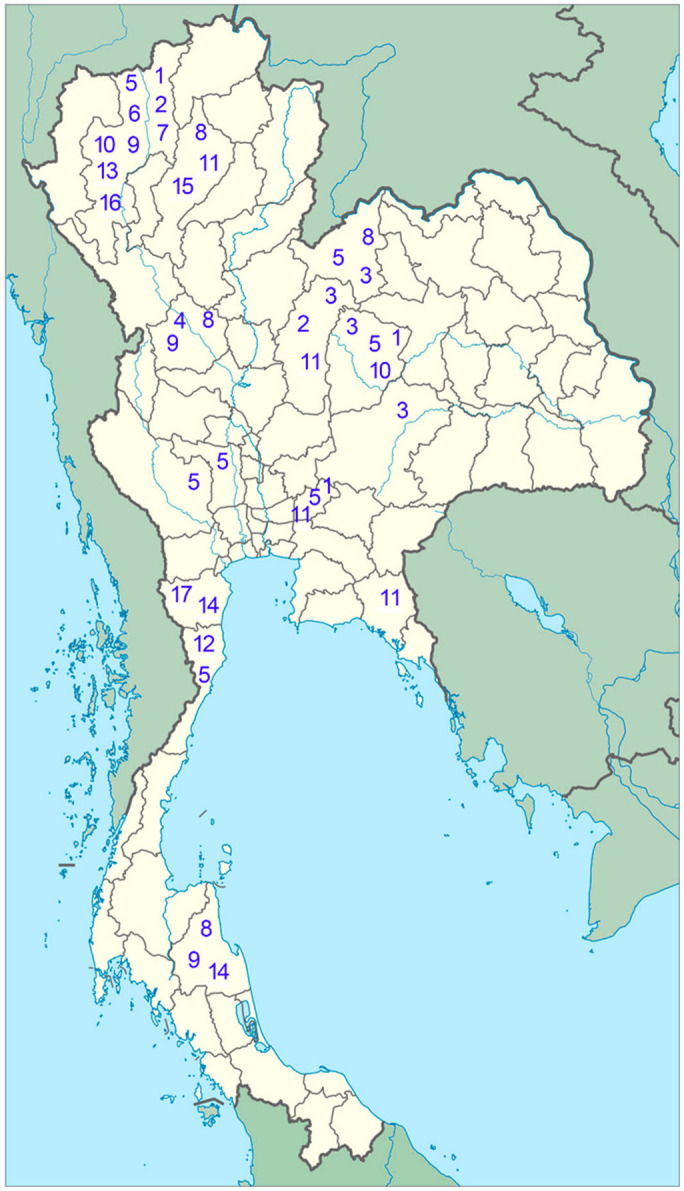
Geographical distributions of *Xestocephalus* species in Thailand. As shown in the figure: **1**. *X. asper*; **2**. *X. guttulatus*; **3**. *X. ishidae*; **4**. *X. gracilus* sp. nov.; **5**. *X. nonattribus* sp. nov.; **6**. *X. binarius* sp. nov.; **7**. *X. densprint* sp. nov.; **8**. *X. toroensis*; **9**. *X. chrysanthemum* sp. nov.; **10**. *X. tenusis* sp. nov.; **11**. *X. exproiecturus* sp. nov.; **12**. *X. abyssinicus*; **13**. *X. cowboyocreus* sp. nov.; **14**. *X. recipinams* sp. nov.; **15**. *X. limpidissimus* sp. nov.; **16**. *X. dimiprocessus* sp. nov.; **17**. *X. malleus* sp. nov.

**Distribution.** Thailand (Petchaburi) (Figure 27).

**Etymology.** The species name is a Latin word that refers to the hammer-like processes of the aedeagal shaft.

**Remarks.** The new species is similar to *X. dimiprocessus* sp. nov. but differs in lacking a long pygofer process and having the aedeagal shaft straight, with basal processes parallel to the shaft and broadened apically (pygofer with long process, aedeagal shaft slightly curved anteriorly, and basal processes tapered to apex in *X. dimiprocessus*).

## 4. Conclusions

Seventeen species of the leafhopper genus *Xestocephalus* Van Duzee from Thailand are reviewed based on a comparative morphological study, including twelve new species and four newly recorded species. As only one species of *Xestocephalus* was previously recorded from Thailand, our study increased the known fauna of this genus in Thailand by 94%. Two of the newly recorded species, described from elsewhere, were previously known to be from China and/or Japan, but two others were previously known to be only from Africa. This suggests that either these species are widespread in the Old World tropics or they have been accidentally introduced from Africa to Thailand (or vice versa). Further study is needed to elucidate the relationship between African and Southeast Asian populations of these species and to determine whether they also occur in other parts of the Old World tropics. At least one other species documented in our study, *X. guttulatus*, appears to be widespread in the Old World and some species belonging to other leafhopper genera (e.g., *Exitianus* Ball, *Balclutha* Kirkaldy, *Cicadulina* China) also have similarly broad distributions. This highlights the need for comprehensive global revisions of such large, widespread genera and the importance of reviewing the taxonomic literature from more than one region when attempting to identify specimens from previously understudied regional faunas.

As in the other large genera of Cicadellidae, less than half of the species of *Xestocephalus* are known only from a few or single localities (Figure 27). Consistent with general worldwide observation, most of the species documented in our study are, so far, only known to be from Thailand. Most of these apparent endemics are recorded from a few or single localities. Nevertheless, four of the new species described from Thailand appear to be more widespread within the country, and, therefore, it seems likely that they will eventually be found in the neighboring parts of Southeast Asia. Further sampling and comparative morphological studies are needed to elucidate the phylogenetic and biogeographic patterns in this large, diverse, and globally distributed genus of leafhoppers.

## Data Availability

Data sharing not applicable.

## Appendix A. Worldwide Checklist of the Genus *Xestocephalus*

Xestocephalus abrotanus Freytag, 2020 Colombia

*Xestocephalus abyssinicus* Heller and Linnavuori, 1968 Democratic Republic of Congo, Ethiopia, Thailand

Xestocephalus acuminatus Freytag, 2020 Colombia

Xestocephalus adiopodoumus dentatus Linnavuori, 1979 Liberia

*Xestocephalus adiopodoumus* Linnavuori, 1979 Ivory Coast

Xestocephalus aethiopicus Melichar, 1914 Tanzania

*Xestocephalus agassizi* Van Duzee, 1912 Jamaica

*Xestocephalus albidus* Evans, 1954 Madagascar

*Xestocephalus albopunctatus* Linnavuori, 1959 Costa Rica, Panama

*Xestocephalus amenus* DeLong, Wolda and Estribi, 1980 Panama

*Xestocephalus ancorifer* Linnavuori, 1959 Brazil, Colombia, Panama, Paraguay

*Xestocephalus angulus* Freytag, 2020 Colombia

*Xestocephalus antimachus* Linnavuori, 1979 Democratic Republic of Congo

*Xestocephalus antlerus* DeLong, Wolda and Estribi, 1980 Panama

*Xestocephalus apicalis* Melichar, 1903 Philippines, Samoa Islands, Sri Lanka

*Xestocephalus aquilus* DeLong, Wolda and Estribi, 1983 Panama

*Xestocephalus artarus* DeLong, Wolda and Estribi, 1980 Panama

*Xestocephalus asper* Linnavuori, 1969 Congo, Thailand

Xestocephalus asper pseudoguttulatus Linnavuori, 1979 Sudan

*Xestocephalus asperus* Freytag, 2020 Colombia

Xestocephalus atratus Kamitani,1996 Japan

Xestocephalus australensis Kirkaldy, 1907 Australia

*Xestocephalus badius* Evans, 1953 Madagascar

*Xestocephalus balli* Van Duzee, 1907 Jamaica

*Xestocephalus baridus* Freytag, 2020 Colombia

*Xestocephalus bicolor* Matsumura, 1914 Japan, China

*Xestocephalus bicoloratus* DeLong, Wolda and Estribi, 1983 Panama

*Xestocephalus bicornis* Linnavuori, 1969 Democratic Republic of Congo, Liberia, Sudan

*Xestocephalus bifasciatus* Cwikla and Wolda, 1986 Panama

*Xestocephalus bifidus* DeLong and Linnavuori, 1978 Mexico

Xestocephalus binarius sp. nov. Thailand

Xestocephalus binatus Cai and He, 2001China

Xestocephalus biprocessus Li and Zhang, 2006 China

Xestocephalus bipunctatus Van Duzee, 1907 Jamaica

Xestocephalus bispinatus Freytag, 2020 Colombia

*Xestocephalus botelensis* Matsumura, 1940 China (Taiwan)

Xestocephalus bulbus Cwikla, 1985 Mexico

*Xestocephalus campsus* Freytag, 2020 Colombia

*Xestocephalus canidia* Linnavuori, 1979 Sudan

*Xestocephalus cervinus* DeLong, Wolda and Estribi, 1980 Panama

*Xestocephalus chibianus* Matsumura, 1940 China (Taiwan)

Xestocephalus chrysanthemum sp. nov. Thailand

Xestocephalus cinctus DeLong, 1980 Peru

Xestocephalus circulus Freytag, 2020 Colombia

Xestocephalus cirus Evans, 1955 Zaire

Xestocephalus clavatus Freytag, 2020 Colombia

Xestocephalus cognatus Choe, 1981 Korea

*Xestocephalus coloreus* Carvalho and Cavichioli 2001 Brazil

*Xestocephalus concolor* Carvalho and Cavichioli 2001 Brazil

*Xestocephalus consentaneus* Linnavuori, 1979 Democratic Republic of Congo, Sudan

Xestocephalus contortuplicatus Kirkaldy, 1907 Fiji

*Xestocephalus coronatus* Osborn and Ball, 1897 United States

Xestocephalus cowboyocreus sp. nov. Thailand

Xestocephalus cristifer Logvinenko, 1981 Russia

*Xestocephalus crocatus* Carvalho and Cavichioli 2001 Brazil

*Xestocephalus culmus* DeLong, Wolda and Estribi, 1980 Panama

*Xestocephalus curtus* DeLong and Linnavuori, 1978 Panama

*Xestocephalus cwiklai* Freytag, 2020 Colombia

*Xestocephalus dedecus* DeLong, Wolda and Estribi, 1980 Panama

Xestocephalus delongi Cwikla, 1985 Jamaica

Xestocephalus densprint sp. nov. Thailand

*Xestocephalus desertorum* (Berg, 1879) Canada to Uruguay

Xestocephalus dimiprocessus sp. nov. Thailand

Xestocephalus dimonika Linnavuori, 1969 Congo

Xestocephalus dubius DeLong, 1982 Bolivia

*Xestocephalus eremnus* Hamitani, 2008 China (Taiwan)

Xestocephalus eumaios Linnavuori, 1973 Cuba

Xestocephalus excertus Freytag, 2020 Colombia

Xestocephalus exproiecturus sp. nov. Thailand

Xestocephalus fasciatus Evans, 1954 Madagascar

*Xestocephalus feowerpacchus* Cwikla, 1985 Anguilla, Antigua, Tortola, St. John

Xestocephalus fistutlus Cwikla, 1985 Panama

*Xestocephalus freyi* (Lindberg, 1936) Canary Islands

*Xestocephalus freytagi* Dmitriev, 2020 Colombia

*Xestocephalus fucatus* Evans, 1954 Madagascar

*Xestocephalus fuliginosus* Kamitani, 2009 West Malaysia

*Xestocephalus fulvus* DeLong, Wolda and Estribi, 1983 Panama

*Xestocephalus fuscarus* DeLong, Wolda and Estribi, 1980 Panama

Xestocephalus fuscomaculatus Kamitani, 2005 Japan

*Xestocephalus fuscus* Evans, 1954 Madagascar

Xestocephalus gracilus sp. nov. Thailand

*Xestocephalus guttulatus* (Motschulsky, 1859) Arabia, China (Taiwan), Ethiopia, Korea, Japan, Malaysia, Philippines, Russia, Saudi Turkey, Sri Lanka, Tanzania, West Indonesia

*Xestocephalus gyrotus* Freytag, 2020 Colombia

*Xestocephalus halimunensis* Kamitani, 2009 Indonesia (Java)

*Xestocephalus huilae* Freytag, 2020 Colombia

*Xestocephalus igerna* Linnavuori, 1969 Congo; Libera

*Xestocephalus iguchii* Matsumura, 1914 Japan, China

*Xestocephalus illus* Freytag, 2020 Colombia

Xestocephalus immaculatus Linnavuori, 1959 Brazil

*Xestocephalus incisus* Freytag, 2020 Colombia

*Xestocephalus irroratus* Osborn, 1924 Argentina, Bolivia, Brazil, Costa Rica, Panama, Paraguay, Peru, West Indies

*Xestocephalus ishidae* Matsumura, 1914 Japan, Thailand

*Xestocephalus izzardi* Metcalf, 1955 Christmas Island, Palau

*Xestocephalus izzardi sodalis* Linnavuori, 1960 Guam, Kosrae, Palau, Pohnpei, S. Mariana Is., Truk, Yap

*Xestocephalus izzardi vuorinoelieae* Lemaître, McKamey and Kment, 2017 Micronesia

*Xestocephalus japonicus* Ishihara, 1961 Japan, Korea

*Xestocephalus javanus* Melichar, 1914:138 West Indonesia: Java

*Xestocephalus jucundus* Linnavuori, 1954 Brazil, Paraguay

Xestocephalus koreanus Kwon, 1981 Korea

Xestocephalus koshunensis Matsumura, 1914 Taiwan

*Xestocephalus kuyanianus* Matsumura, 1914 Taiwan, Japan

*Xestocephalus latus* Freytag, 2020 Colombia

*Xestocephalus lentus* Freytag, 2020 Colombia

*Xestocephalus ligatus* Freytag, 2020 Colombia

Xestocephalus limpidissimus sp. nov. Thailand

Xestocephalus longipilus Freytag, 2020 Colombia

*Xestocephalus longus* Cwikla, 1985 Cayman Islands

*Xestocephalus lunatus* Peters, 1933 United States

Xestocephalus lunulatus Freytag, 2020 Colombia

*Xestocephalus luridus* Linnavuori, 1959 Costa Rica, Panama

*Xestocephalus maculatus* Osborn, 1929 Cuba, Puerto Rico

Xestocephalus magdaleniensis Freytag, 2020 Colombia

Xestocephalus magnificus Evans, 1966 Australia

*Xestocephalus magnus* Freytag, 2020 Colombia

Xestocephalus malleus sp. nov. Thailand

Xestocephalus maquilingensis Merino, 1936:387 Philippines

Xestocephalus mectopilus Freytag, 2020 Colombia

*Xestocephalus medius* Linnavuori, 1969 Congo

*Xestocephalus mexicanus* DeLong and Linnavuori, 1978 Mexico

*Xestocephalus mimicus* Freytag, 2020 Colombia

*Xestocephalus minimus* China, 1935 West Indonesia: Sumatra

*Xestocephalus miramari* DeLong, Wolda and Estribi, 1980 Panama

*Xestocephalus mirus* Freytag, 2020 Colombia

*Xestocephalus montanus* Matsumura, 1914 China (Taiwan)

*Xestocephalus nanus* Freytag, 2020 Colombia

*Xestocephalus nastus* Freytag, 2020 Colombia

*Xestocephalus nemus* Freytag, 2020 Colombia

Xestocephalus neobadius Dmitriev 2020 Panama

*Xestocephalus nigrus* Freytag, 2020 Colombia

*Xestocephalus nikkoensis* Matsumura, 1914 Japan, China

Xestocephalus nilgiriensis Distant, 1918 India

Xestocephalus nonattribus sp. nov. Thailand

Xestocephalus obscurusHamitani,1996 Japan

*Xestocephalus obtusus* Freytag, 2020 Colombia

*Xestocephalus ornatus* Van Duzee, 1907 Jamaica

*Xestocephalus osborni* Merino, 1936 Philippines

*Xestocephalus ovalis* Evans, 1966 New Zealand

*Xestocephalus paganurus* Melichar, 1903 Philippines, Samoa, Sri Lanka

Xestocephalus palintus Freytag, 2020 Colombia

*Xestocephalus pallescens* Linnavuori, 1979 Democratic Republic of Congo

Xestocephalus pallidiceps Kirkaldy, 1907 Fiji

*Xestocephalus panamanus* DeLong, Wolda and Estribi, 1983 Panama

*Xestocephalus parvus* Freytag, 2020 Colombia

Xestocephalus pianmaensis Li and Dai,2005 China

Xestocephalus piceatus Osborn, 1934 Fiji

*Xestocephalus polleti* Linnavuori, 1979 Ivory Coast, Nigeria

*Xestocephalus primus* Freytag, 2020 Colombia

*Xestocephalus pullus* DeLong, Wolda and Estribi, 1983 Panama

*Xestocephalus punctatus* Caldwell, 1952 Puerto Rico

*Xestocephalus punctulatus* Carvalho and Cavichioli 2001 Brazil

*Xestocephalus purpurascens* Kirkaldy, 1907 Australia, Fiji

Xestocephalus quadratus Freytag, 2020 Colombia

Xestocephalus quadripunctatus Linnavuori, 1955 Brazil

*Xestocephalus ramulus* DeLong and Linnavuori, 1978 Puerto Rico

Xestocephalus recipinams sp. nov. Thailand

Xestocephalus reflexus Osborn, 1934 Samoa

*Xestocephalus relatus* Distant, 1918 India, Philippines

Xestocephalus ryukyuensis Kamitani, 2005 Japan

*Xestocephalus sidnicus* Kirkaldy, 1907 Australia

Xestocephalus similus DeLong, 1982 Brazil

Xestocephalus sinchonus DeLong, 1982 Peru

*Xestocephalus spicatus* DeLong and Linnavuori, 1978 Mexico

Xestocephalus spinatus Freytag, 2020 Colombia

*Xestocephalus spinestyleus* Li and Dai, 2003 China (Taiwan)

*Xestocephalus spinifer* Linnavuori, 1979 Congo, Liberia, Tanzania

*Xestocephalus spinosus* Linnavuori, 1957 Solomon Isl.

*Xestocephalus stellatus* Carvalho and Cavichioli 2001 Brazil

*Xestocephalus suakoko* Linnavuori, 1979 Liberia

Xestocephalus subfusculus Melichar, 1905 Tanzania

*Xestocephalus subtessellatus* Linnavuori, 1959 Costa Rica, Panama

Xestocephalus sycophantus Linnavuori, 1979 Cameroon

*Xestocephalus takahashii* Kamitani, 2009 West Malaysia

*Xestocephalus talus* Freytag, 2020 Colombia

*Xestocephalus tangaensis* Linnavuori, 1979 Tanzania

Xestocephalus tasmaniensis Evans, 1938 Australia

Xestocephalus tenusis sp. nov. Thailand

*Xestocephalus tessellatus* Van Duzee, 1894 Bolivia, Brazil, Colombia, Costa Rica, Cuba, Panama, United States

*Xestocephalus tetracerus* Kamitani,2009 Indonesia (Java).

*Xestocephalus toroensis* Matsumura, 1914 China (Taiwan), Japan, Thailand

Xestocephalus transversus Distant, 1918 India

*Xestocephalus triatus* Caldwell, 1952:40 Puerto Rico

Xestocephalus trigonus Freytag, 2020 Colombia

*Xestocephalus tripartitus* Carvalho and Cavichioli 2001 Brazil

*Xestocephalus tucsoni* Knull, 1944 United States

Xestocephalus tutuilanus Osborn, 1934 Fiji, Samoa

Xestocephalus uncinatus Freytag, 2020 Colombia

Xestocephalus undulatus Freytag, 2020 Colombia

Xestocephalus variarius DeLong, 1982:26 Peru

Xestocephalus vitiensis Kirkaldy, 1907:51 Fiji

Xestocephalus vitiensis mancus Linnavuori, 1960:34 Fiji

Xestocephalus vitiensis triceros Linnavuori, 1960:33 Fiji

*Xestocephalus vittanotus* Cwikla and Wolda, 1986:344 Panama

*Xestocephalus youngi* Cwikla, 1985:214 Cuba

*Xestocephalus zambicus* Linnavuori, 1979:945 Democratic Republic of Congo
